# Species Identity Drives Geographic Variation in Leaf Traits and Palatability to a Generalist Insect Herbivore of a Common Invader

**DOI:** 10.1002/ece3.74387

**Published:** 2026-09-23

**Authors:** Katie Lee, Stacy B. Endriss, Bernd Blossey

**Affiliations:** ^1^ Department of Natural Resources and the Environment Cornell University Ithaca New York USA; ^2^ Department of Entomology Virginia Tech Blacksburg Virginia USA

**Keywords:** functional traits, herbivory, hybridization, knotweeds, latitudinal clines, *Reynoutria*

## Abstract

Introduced plants represent unique opportunities to understand how diverse phenotypic traits vary across broad geographic scales. However, clear generalizable principles about genetically based variation in key functional plant traits across geographic clines remain elusive. For example, functional plant traits often vary predictably across climatic gradients, yet these emergent patterns may be obscured by other factors that may collectively shape trait expression across a species' range, such as species‐level differences or understudied non‐climatic factors that operate across latitude. To address this knowledge gap, we leveraged an established common garden of 213 individuals of the octoploid *Reynoutria japonica* and the hybrid polyploid *R.* × *bohemica* initially collected over a wide latitudinal range across the eastern United States. Specifically, we assessed how heritable variation in *Reynoutria* spp. resistance to herbivory, measured as performance of the generalist herbivore *Spodoptera exigua* and leaf traits affecting herbivory, may be explained by climate, latitude, or interspecific differences. Interspecific differences, such as those mediated by hybrid polyploidy, were the main factors explaining variation in resistance to herbivory: *R. japonica* was generally more resistant and less palatable than *R*. × *bohemica*. *Spodoptera exigua* performance and leaf trait variation were also better explained by models that included climate rather than latitude at original collection locations. Herbivore survival to adult increased when feeding on *R. japonica* and decreased when feeding on *R*. × *bohemica* as climates at collection locations became colder and more seasonally variable. Plants originating from wetter and more seasonally consistent climates also had tougher leaves than plants from more seasonally variable climates. Our findings implicate the critical role of interspecific differences, potentially mediated by linked evolutionary mechanisms, in shaping heritable variation in resistance to herbivory across species' introduced ranges. We found that climate also mediates trait expression and herbivore performance, but its effects were less prominent than those associated with interspecific differences between two closely related congeners.

## Graphical abstract

Introduced plants represent unique opportunities to understand how diverse phenotypic traits vary across broad geographic scales. However, clear generalizable principles about genetically based variation in key functional plant traits across geographic clines remain elusive. To address this knowledge gap, we assessed how heritable variation in knotweed resistance to herbivory, measured as performance of a generalist herbivore and leaf traits affecting herbivory, may be explained by climate, latitude, or interspecific differences.

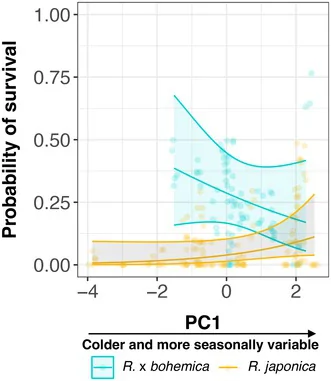

## 1 Introduction

Plants can express predictable heritable, phenotypic variation in traits that mediate key emergent patterns in ecosystem function across abiotic and biotic gradients (Fridley and Grime 2010; Hughes et al. 2008; Kraft et al. 2014; Siefert et al. 2015). Indeed, the ability to predict complex ecosystem processes from species' traits has been described as a “Holy Grail” in ecology (Funk et al. 2017; Lavorel and Garnier 2002). Although generalizable patterns in trait variation may exist, they may only be disentangled after accounting for the diverse factors that shape heritable variation across a species' range (Hofhansl et al. 2021; van der Plas et al. 2020; Violle et al. 2007). Further, even when emergent patterns are revealed, how predictable variation in key functional traits cascades to shape species interactions and ecosystem functioning remains poorly understood, in part because the traits most directly targeted by selection are often overlooked (Funk et al. 2017; van der Plas et al. 2020). Thus, the underlying factors mediating predictable, genetically based trait variation and eco‐evolutionary dynamics across broad geographic scales remain critical knowledge gaps.

Introduced plant species are generally well suited for studying the factors that best explain genetically based trait variation, especially in functional traits related to resistance against herbivory. As plants often “escape” herbivory (especially by their co‐evolved specialist herbivores) upon introduction to a new range (Keane and Crawley 2002), selection on traits related to defense may weaken, enabling selection pressures such as climate and other environmental factors that vary across latitudinal clines, such as resource competition, to play a more prominent role in shaping evolutionary trajectories (Blossey and Nötzold 1995; Endriss et al. 2022; Woods and Sultan 2022; Zhang et al. 2018). Furthermore, introduced species are often distributed across broad geographic ranges (Colautti and Barrett 2013; Maron et al. 2004; van Boheemen et al. 2019), and increasing evidence suggests that, contrary to historical assumptions, populations can rapidly adapt to novel environments as frequently and rapidly in their introduced as in their native ranges (Battlay et al. 2025; Oduor et al. 2016). Thus, introduced species represent unique opportunities to investigate how climate and other evolutionary factors structure predictable phenotypic variation and species interactions across geographic scales.

Variation in traits related to resistance against herbivory is often investigated along latitudinal clines, as latitude is a composite proxy for multiple underlying environmental gradients (Willig et al. 2003). Indeed, herbivore pressure or defenses against herbivory may depend upon abiotic drivers that track latitudinal clines such as temperature and seasonality (Moreira et al. 2015). At lower latitudes, more favorable abiotic environments can promote higher species richness among both host plants and insect herbivores (Willig et al. 2003). As species interactions intensify toward the tropics, herbivore pressure, and thus selection for plant defenses such as trichomes (Kooyers et al. 2015) and secondary metabolites (Anstett et al. 2015; Pratt et al. 2014; Rasmann and Agrawal 2011), are predicted to increase in low‐latitude communities (Coley and Aide 1991). However, evidence for this hypothesis remains mixed across ecosystems, such that clear generalizable predictions about leaf herbivore‐resistance traits across latitudinal clines remain elusive (Loughnan and Williams 2019). Some studies report stronger herbivory at higher latitudes (Adams and Zhang 2009; Lehndal and Ågren 2015; Salgado and Pennings 2005) or near the center of a species' range (Woods et al. 2012), while others find no clear generalizable patterns (Andrew and Hughes 2005; Anstett et al. 2018; Klingaman and Oliver 1996). Such discrepancies in the presence or direction of latitudinal trends have been attributed in part to the frequent use of latitude as an imperfect proxy for climatic variation in establishing theory and hypotheses (Hahn and Maron 2016). Other processes can also act across latitudinal clines, such as accumulation of genetic mutations, genetic drift, or local adaptation to non‐climatic correlates of latitude (Colautti et al. 2009; Croy et al. 2022; Dlugosch et al. 2015). Moreover, certain direct measures of climate, such as precipitation, may not correlate predictably with latitude, further complicating efforts to draw broad latitudinal inferences.

Growing evidence suggests that explicitly examining the effects of climate, rather than using latitude as a proxy, provides more robust inference into the factors structuring geographical variation in plant herbivore‐resistance traits (Hahn and Maron 2016; Moreira et al. 2015). Climate may mediate the evolution of these traits both directly—by imposing physiological constraints or favoring specific trait syndromes (Wright et al. 2004)—and indirectly—by influencing herbivore community composition, intensity or type of herbivory, or resource availability and, in turn, altering their relative selective pressure (Loughnan and Williams 2019; Maron et al. 2014; Moreira et al. 2015). By influencing the availability of resources to plants, local climatic conditions can drive regional variation in plant growth and leaf traits (Wright et al. 2004): resource availability may be lower in colder regions where tissue loss is more costly, causing plants to evolve slower growth rates and greater investment in defense, whereas species in resource‐rich environments allocate more resources to regrowth at the expense of resistance to herbivory (Coley et al. 1985). However, these mechanisms are not mutually exclusive, and empirical support exists for both strategies across diverse ecosystems (Anstett et al. 2018). Thus, spatially variable selective pressures can generate complex patterns of plant resistance that are difficult to generalize across broad climatic gradients (Loughnan and Williams 2019).

In addition to climate, plants vary greatly in their level and mechanism of resistance, among both species and genotypes within species (Kempel et al. 2011). These species‐specific differences in plant growth and defense may be mediated by commonly linked evolutionary mechanisms, such as interspecific hybridization and polyploidy (i.e., the doubling of chromosome sets) (Assour et al. 2024; Boecklen and Spellenberg 1990; Halverson et al. 2008; Krebs et al. 2011; Thompson et al. 2004), and have been implicated in numerous plant invasions (Schierenbeck and Ellstrand 2009). Hybridization can produce phenotypes with levels of resistance to herbivory that are intermediate between parental species (Aguilar and Boecklen 1992; Fritz et al. 1996), closely resemble one parent (Fritz et al. 1996; Whitney et al. 2006), or are better adapted to particular environments than either parent and may therefore be favored by natural selection (Krebs et al. 2011; Rieseberg et al. 2003). The latter may occur when hybridization followed by segregation generates novel recombinant genotypes, resulting in either enhanced (Boecklen and Spellenberg 1990) or reduced resistance to herbivory (Fritz et al. 1994, 1996; Whitham et al. 1999). Hybridization can also drive both gene recombination and changes in ploidy (i.e., the number of sets of chromosomes) that collectively may shape functional traits and, in turn, how species respond to abiotic and biotic stressors (Assour et al. 2024; Gross and Schiestl 2015; Segraves and Anneberg 2016; Wei et al. 2019). Interspecific hybridization that results in a shift in ploidy through whole genome duplication (hereafter, “hybrid polyploidy”), for example, has been shown to drive meaningful differences in plant size, trichome density, leaf thickness, and secondary metabolite production (Bomblies 2020; Hamarashid et al. 2022; Wei et al. 2019). Hybrid polyploidy may also interact with selection pressures such as climate to further influence trait expression, resulting in genetically based, population‐level trait differences associated with the climate of the latitude where populations evolved (Meyerson et al. 2016; Williams et al. 2014). Thus, understanding the extent to which species‐level differences—especially those mediated by linked genetic mechanisms such as hybridization and polyploidy—explain evolutionary patterns in resistance traits and plant‐herbivore interactions across diverse novel environments remains a critical area of research (Martin and Husband 2009; Segraves and Anneberg 2016; Suda et al. 2015; Williams et al. 2014).

Here, we use the globally invasive Japanese knotweed ( *Reynoutria japonica* ) and Bohemian knotweed (*R*. × *bohemica*) to assess how genetically based variation in resistance to herbivory, measured in terms of performance of a generalist herbivore and leaf herbivore‐resistance traits, can be explained by climate, latitude, or interspecific differences across their introduced North American range. To this end, we leveraged an established experimental common garden of *R. japonica* and the hybrid polyploid *R*. × *bohemica* representative of a wide latitudinal range (~11° of latitude, 1761 km) across the eastern United States.

Specifically, we tested whether (1) genetically based variation in resistance to herbivory can be better explained as a function of species identity (i.e., *R. japonica* vs. *R*. × *bohemica*) compared to climatic or latitudinal clines. We then explored whether (2) climate or latitude (as a composite proxy for multiple environmental gradients) at the original collection locations is a superior explanation of genetically based variation in resistance to herbivory. We assessed this variation using two approaches: (1) insect herbivore performance (i.e., pupal and adult weights, survival, and development time to the pupal and adult stages) and (2) leaf traits affecting herbivory (i.e., leaf toughness and specific leaf area [SLA]). We chose leaf toughness and SLA as key structural leaf traits commonly linked to variation in insect herbivory (Hanley et al. 2007; Zhao et al. 2024). Tougher leaves are generally more difficult for herbivores to tear or penetrate (Zhao et al. 2024). SLA is often positively correlated with relative growth rate but negatively correlated with leaf thickness, such that leaves with low SLA tend to have denser, less palatable tissue often associated with reduced performance of herbivores (Kozlov et al. 2015; Zhao et al. 2024).

## 2 Materials and Methods

### 2.1 Study System

*Reynoutria japonica* (Houtt) was introduced as an ornamental from Japan to Europe in the 1840s, and within a few decades to North America (Barney 2006; Del Tredici 2017). Most introduced *R. japonica* populations are thought to have originated from a single 8*x* genotype (Gaskin et al. 2014; Groeneveld et al. 2014; Richards et al. 2012) or a very small founding genetic pool (Burns et al. 2025). However, *R. japonica* (typically 2*n* = 8*x* = 88) can hybridize with giant knotweed ( *R. sachalinensis* [F. Schmidt] Nakai) (typically 2*n* = 4*x* = 44), resulting in an introduced knotweed species complex composed of *R. japonica* , *R. sachalinensis* , and their hybrid polyploid *R*. × *bohemica* (Chrtek and Chrtková) (typically 2*n* = 6*x* = 66) (hereafter, “knotweeds”). *Reynoutria sachalinensis* is less abundant, but *R. japonica* and *R*. × *bohemica* are widely distributed across their introduced North American range, with the hybrid polyploid being the most genetically diverse knotweed species (Gaskin et al. 2014). Recent molecular and historical analyses further indicate multiple introductions (Del Tredici 2017; Tippery et al. 2025), and mechanisms such as hybridization, introgression, and polyploidy may enhance genetic variation (Tippery et al. 2021), both at the individual species level and within the species complex as a whole, increasing potential for local adaptation of knotweeds following their initial introduction to North America. Knotweed is considered one of the world's most invasive species (Lowe et al. 2000), forming near‐monocultures especially in riparian and disturbed habitats (Barney et al. 2006) that suppress native understory plants and degrade habitat for soil and aquatic invertebrates and amphibians (Brousseau et al. 2021; Claeson et al. 2014; Maerz et al. 2005; Siemens and Blossey 2007).

Knotweeds experience significant enemy release in their introduced North American range (Irimia et al. 2025), resulting in, at present, failed attempts to establish the Japanese psyllid (*Aphalara itadori*) for biological control (Grevstad et al. 2025). Adults of the non‐native generalist Japanese beetle ( *Popillia japonica* ) can be a common, albeit temporary, leaf feeder of knotweeds in late summer (Johnson et al. 2019). However, their attack is insufficient to reduce performance or initiate demographic declines in knotweed populations; thus, we do not investigate either herbivore as a driver of observed variation in herbivore‐resistance traits.

### 2.2 Experimental Approach

We used an outdoor common garden of both *R. japonica* and *R*. × *bohemica* established in 2019 at the Cornell Resource Ecology and Management Facility (REM) in Ithaca, NY (42.4427° N, 76.4692° W) to investigate potential factors explaining genetically based variation in knotweed resistance to herbivory (i.e., herbivore performance and leaf traits). Our common garden was initiated using two to six knotweed propagules collected from each of 46 locations ranging from Georgia to Maine (Figure S1; Table S1). We established each propagule from rhizome cuttings that were thoroughly washed and cleaned until no soil remained, then individually transplanted into 12 L pots in 2019, and later into 100 L tree pots in 2020 (BFG Supply, Lancaster, New York, USA). We grew plants in a 1:1:1 medium of sand (RMS Gravel, Dryden, New York, USA), compost (WeCare Organics, Woodbind, Maryland, USA), and screened topsoil (Reeves Brother Farms, Lansing, New York, USA). Genetic analyses indicate that *R. japonica* plants in our common garden mostly share the same chloroplast haplotype as populations native to Japan, indicating both Japan as their likely origin and that limited among‐population variation exists at the haplotype level (Wang et al. 2025; Zhang et al. 2024).

### 2.3 Herbivore Performance: Weight, Survival, Development Time

We chose to use the cosmopolitan generalist beet armyworm ( *Spodoptera exigua* ; Lepidoptera: Noctuidae) to test whether genetically based variation in knotweed leaf traits impacts herbivore performance. We chose *S. exigua* because the species is often used as a model organism in herbivore studies (Szulińska et al. 2025), the species is known to feed on knotweeds in parts of the plants' native distribution (Zhao et al. 2024), and there is a lack of knotweed herbivores in North America (Grevstad et al. 2018). We obtained *S. exigua* eggs (Benzon Research, Carlisle, Pennsylvania, USA) in summer 2022, allowed them to hatch, and within 24 h we transferred individual neonates into portion cups (30 mL; Dart Container, Mason, Michigan, USA). Each cup contained young knotweed leaf tissue positioned on moist filter paper (47 mm, Grade 1; Whatman, Buckinghamshire, UK). We used leaf material from 46 collection locations, choosing one plant per collection location (29 *R. japonica* , 17 *R*. × *bohemica*) and established five replicates per plant (*N* = 230 cups).

We assessed the quality of our insect population at the start of the experiment using an artificial diet (Benzon Research, Carlisle, Pennsylvania, USA). We stored diet in a walk‐in cooler (6°C) for 2 days before transferring 10 individual neonates—one per cup—into portion cups, each containing 20 mL of artificial diet. We sealed cups—including the lids—with Parafilm to prevent insect escape until they reached second instars, at which point we removed Parafilm to allow for ventilation. A total of seven individuals (70%) completed development to the pupal stage.

For the 48‐day duration of the experiment, we placed all portion cups under grow lights across five shelves of a shelving unit in a walk‐in growth chamber with a 16‐h photoperiod and 25°C ± 1°C consistent temperatures. For the first 2 days of the experiment, we replaced all dead or missing larvae with new neonate individuals. We refreshed leaf material as needed to ensure that all individuals were satiated throughout the experiment and haphazardly rearranged portion cups within each shelf every 3 days. We monitored *S. exigua* and scored survival every 2 days until death or adult emergence. We weighed (±0.0001 g; Model BP211D, Mettler Toledo, Columbus, Ohio, USA) individual pupae and adults on the first day we observed their pupation and adult emergence, respectively.

### 2.4 Knotweed Leaf Traits: Leaf Toughness, SLA

In August 2020, we thoroughly watered all plants in our existing common garden to rehydrate leaves and ensure maximum turgidity. The next day, we sampled two leaves from one stem from each plant, prioritizing leaves that were relatively undamaged by the generalist *P. japonica* . We cut one young (first fully expanded leaf produced after peak *P. japonica* herbivory) and one older leaf (produced before peak *P. japonica* herbivory) at the base of their petioles to assess potential effects of leaf age as a proxy for leaf production relative to peak *P. japonica* herbivory on the leaf traits measured here.

To measure leaf toughness, we clamped each leaf between two 1 mm‐thick rubber pads adhered to wooden plates 34.5 × 7.3 × 0.8 cm (*L* × *W* × *H*), each with aligned 7.1 mm‐diameter guide holes. We used a digital push‐pull force gauge penetrometer (Model M2‐2, Mark‐10, Copiague, New York, USA) with a maximum capacity of ~9.81 Newtons (N) (accurate to within 0.5% of the force measurement) and 6.4 mm punch rod diameter to determine the maximum force required to puncture the apex, middle, and base of each leaf, immediately adjacent to the left of the midrib (0.35 mm clearance between edges of the punch and hole). We averaged values from each position within the leaf (apex, middle, base) and leaf age (young, older) to estimate leaf toughness for each plant.

To measure SLA, we hole punched two 6 mm‐diameter leaf discs (one each from near the apex and from near the base) from the right side of the same leaves. Each disc therefore had a leaf surface area of 28 mm^2^ as calculated using the area formula (i.e., area = *π* × radius^2^). We stored leaf discs in a walk‐in cooler (4°C for 24 h), then oven‐dried (45°C for 24 h) and weighed them (±0.0001 g; Model BP211D, Mettler Toledo, Columbus, Ohio, USA). We averaged values of SLA across positions within the leaf (apex, base) and leaf ages (young, older) to generate a single estimate per plant. We averaged leaf trait measurements both across leaf ages and positions within the leaf as we did not observe significant effects of either on leaf toughness or SLA in our preliminary analyses.

### 2.5 Statistical Analyses

We conducted all analyses in R v.4.4.3 (R Core Team 2025). We used the *lmer* function in the package lme4 (Bates et al. 2015) to run linear mixed models (LMMs) and generalized linear mixed models (GLMMs). For all analyses, we used the *dredge* function in the MuMIn package (Bartoń 2022) to run model selection to determine the most parsimonious models overall according to corrected Akaike information criterion (AICc) and to estimate each model's conditional *R* ^2^ (Table 1). We reported a subset of the most parsimonious models that included latitude (Table S2) as well as all models with ΔAICc < 2 from the most parsimonious model (i.e., top models; Tables S3–S10) (Burnham and Anderson 2002). Prior to conducting analyses, we used the car package (Fox and Weisberg 2019) to confirm absence of strong collinearity between all possible main effects (i.e., VIF < 5) (Zuur et al. 2009). We used the *emmeans* and *emtrends* functions in the emmeans package (Lenth 2022) to run post hoc tests with Kenward‐Roger adjusted degrees of freedom (Kenward and Roger 1997) on the best (i.e., most parsimonious) models for factors identified as significant by type III analysis of variance (ANOVA, *p* < 0.05). To characterize variation in knotweed leaf traits within collection location, we calculated descriptive statistics (i.e., mean, standard deviation, minimum, and maximum) across sampled plants for each collection location (Table S11). Because we assessed herbivore performance using only one plant per collection location, we could not quantify within‐location variation in herbivore performance among plants.

**TABLE 1:** Summary of the best models (defined as models with the lowest AICc values) fit to data of herbivore performance (gray rows) and knotweed leaf traits (orange rows).

Response	Best model	*R* ^2^	*w*_i_	Model interpretation	Figures, Tables
Survival (pupa)	**Species** + PC3	0.245	0.438	Survival to pupal stage was lower for *R. japonica* than for *R*. × *bohemica* (*p* < 0.001)	Figure 1, Table S3
Survival (adult)	**Species** + PC1 + **species** * **PC1** + PC3	0.323	0.159	Survival to adult stage was higher for *R*. × *bohemica* than for *R. japonica* in warmer and more seasonally consistent climates; however, survival increased for *R. japonica* , and decreased for *R*. × *bohemica*, as climates at collection locations became colder and more seasonally variable (*p* = 0.047)	Figure 4, Table S4
Pupal weight	~1 (intercept‐only model)	0.032	0.728	None of our measured variables explained the variation in our data better than an intercept‐only model	Table S5
Adult weight	PC3	0.193	0.498	Adult weight decreased as climates at collection locations became more seasonally consistent, but was not significant across the climatic gradient (*p* = 0.10)	Table S6
Development time to pupal stage	**Species**	0.348	0.372	Development to the pupal stage took longer for *R. japonica* than for *R*. × *bohemica* (*p* < 0.001)	Figure 2a, Table S7
Development time to adult stage	**Species**	0.260	0.627	Development to the adult stage took longer for *R. japonica* than for *R*. × *bohemica* (*p* < 0.01)	Figure 2b, Table S8
Leaf toughness	**Species** + **PC1** + **PC2** + PC3 + species * PC3	0.354	0.458	Leaf toughness was higher for *R. japonica* than for *R*. × *bohemica* (*p* < 0.001) and increased as climates at collection locations became wetter and more seasonally consistent (Figure 5)	Figure 3a, Figure 5, Table S9
SLA	**Species**	0.373	0.219	SLA was lower for *R. japonica* than for *R*. × *bohemica* (*p* < 0.001)	Figure 3b, Table S10

*Note:* Models of knotweed traits included collection location ID, or collection location ID and shelf for models of herbivore performance, as random intercepts. Significant main and interactive effects (*p* < 0.05) in each model are bolded, and their marginal means or trends calculated using *emmeans* and *emtrends* as discussed in the model interpretation column. PC1, PC2, and PC3 represent three linear principal components (PCs) related to climate. PC1 and PC2 were both attributed mainly to temperature and precipitation. As values of PC1 increase, climates at collection locations are colder and have larger annual temperature ranges. As values of PC2 increase, climates at collection locations are colder, wetter, and precipitation is more seasonally consistent. In contrast, PC3 was attributed mainly to climate seasonality: as values of PC3 increase, monthly temperature ranges become smaller and precipitation is more seasonally consistent.

To examine effects of climate on herbivore performance and knotweed leaf traits, we extracted from WorldClim (www.worldclim.org) 19 bioclimatic variables derived from monthly climate data (i.e., temperature, precipitation, and their variability for the 1970–2000 period) for the collection location of each of our 213 knotweed plants. We excluded highly correlated bioclimatic variables (Pearson's correlation coefficient [*r*] > 0.7) to improve interpretability. Within each pair of highly correlated variables, we retained, when possible, the variable that was most ecologically relevant and reflected climatic conditions over time. Overall, we retained seven variables: bio2 (mean diurnal range), bio7 (temperature annual range), bio8 (mean temperature of wettest quarter), bio9 (mean temperature of driest quarter), bio10 (mean temperature of warmest quarter), bio12 (annual precipitation), and bio15 (precipitation seasonality) (see Figure S2 for more detailed description of each bioclimatic variable). We performed hierarchical clustering using Pearson correlations as well as principal component analysis (PCA) on these variables (standardized and centered) using the *prcomp* function, and extracted principal components (PCs) with eigenvalues > 1 (based on the Kaiser‐Guttman criterion; Kaiser 1960) for downstream analyses (Figure S2; Table S12).

PC1 and PC2 were both attributed mainly to temperature and precipitation (Figure S2; Table S12). As values of PC1 increase, climates at collection locations are colder and have larger annual temperature ranges. As values of PC2 increase, climates at collection locations are colder, wetter, and precipitation is more seasonally consistent. In contrast, PC3 was attributed mainly to climate seasonality: as values of PC3 increase, monthly temperature ranges become smaller and precipitation is less seasonally variable (Figure S2; Table S12). The first three PCs cumulatively explained 82.3% (37.4%, 28.3%, and 16.7%, respectively) of the climatic variation across the collection locations of each of our knotweed plants (Figure S2).

We fit GLMMs (binomial) to survival data in three ways to assess whether interspecific differences, climate, or latitude at collection location best explains variation in herbivore performance: (1) using all possible combinations of the fixed effects of the three climate PCs, species ( *R. japonica* vs. *R*. × *bohemica*), and all possible two‐way interactions between each PC and species; (2) using a similar set of GLMMs, but with all possible combinations of latitude (instead of the climate PCs), species, and a two‐way interaction between latitude and species. We also fit a set of LMMs to data of pupal weight, adult weight, and development time to pupa and adult. We chose to compare competing models containing climate versus latitude as fixed effects, as latitude and climate PC1 were highly correlated (*r* = 0.96). As latitude was not correlated with either climate PC2 (*r* = 0.17) or PC3 (*r* = −0.06), we also investigated their relative contribution within the same models by replacing climate PC1 with latitude in all models: (3) we fit all possible combinations of the fixed effects of latitude (instead of climate PC1), species, climate PCs 2 and 3, and all possible two‐way interactions between species and latitude, and between species and each PC. We included collection location ID and shelf as random intercepts in all models of herbivore performance.

We fit a set of candidate LMMs to data of leaf toughness and SLA (log‐transformed), again in three ways, to assess whether interspecific differences, climate, or latitude at collection location best explains variation in knotweed herbivore‐resistance traits: (1) using all possible combinations of the fixed effects of the three climate PCs, species, and all possible two‐way interactions between species and each PC; (2) using a similar set of LMMs, but with all possible combinations of latitude (instead of the climate PCs), species, and a two‐way interaction between species and latitude; (3) using all possible combinations of the fixed effects of latitude (instead of climate PC1), species, climate PCs 2 and 3, and all possible two‐way interactions between species and latitude, and between species and each PC. We included collection location ID as a random intercept effect in all models of leaf traits. We report all models using latitude in place of climate PC1 with ΔAICc < 2 from the most parsimonious model (Burnham and Anderson 2002) in Tables S13–S20.

## 3 Results

**FIGURE 1:**
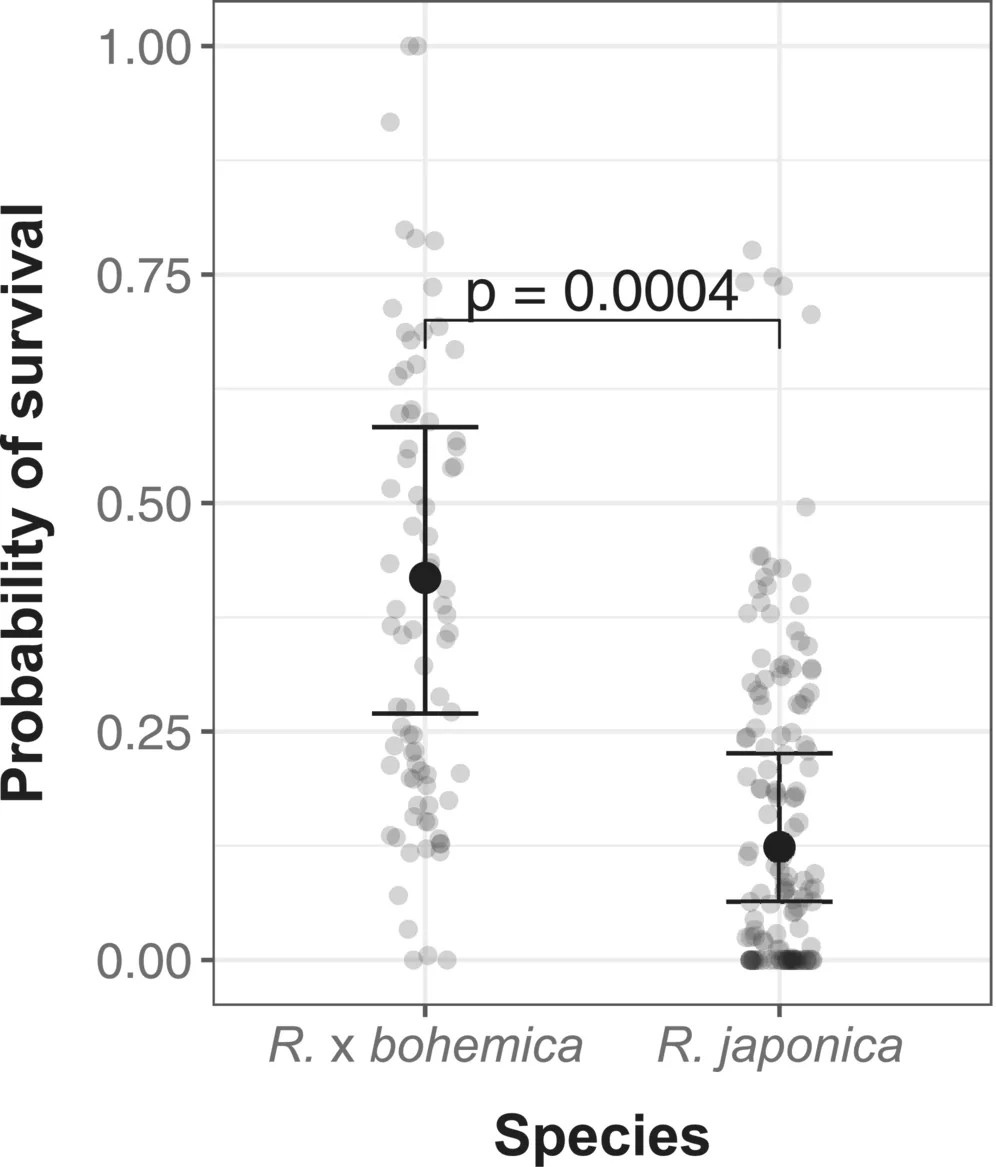
Probability of *S. exigua* survival to pupa when fed *R*. × *bohemica* or *R. japonica* . Semi‐transparent points are raw data, while larger points and error bars depict model‐predicted estimated marginal means and 95% CI from our best GLMM of survival to pupa (Table 1).

**FIGURE 2:**
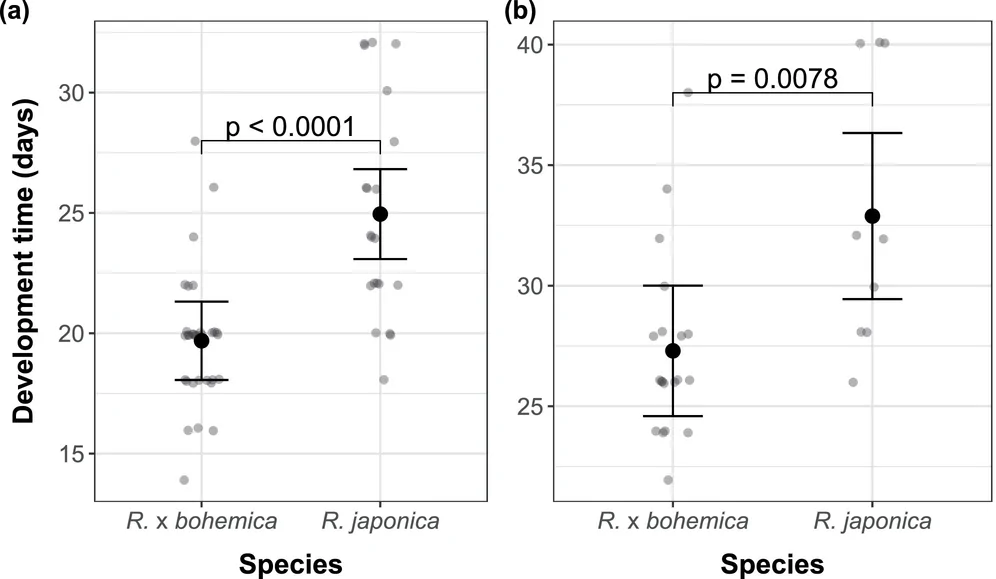
Development time (days) of *S. exigua* to (a) pupa and (b) adult when fed *R*. × *bohemica* or *R. japonica* . Semi‐transparent points are raw data, while larger points and error bars depict model‐predicted estimated marginal means and 95% CI from our best LMMs of development time to pupa and adult, respectively (Table 1).

**FIGURE 3:**
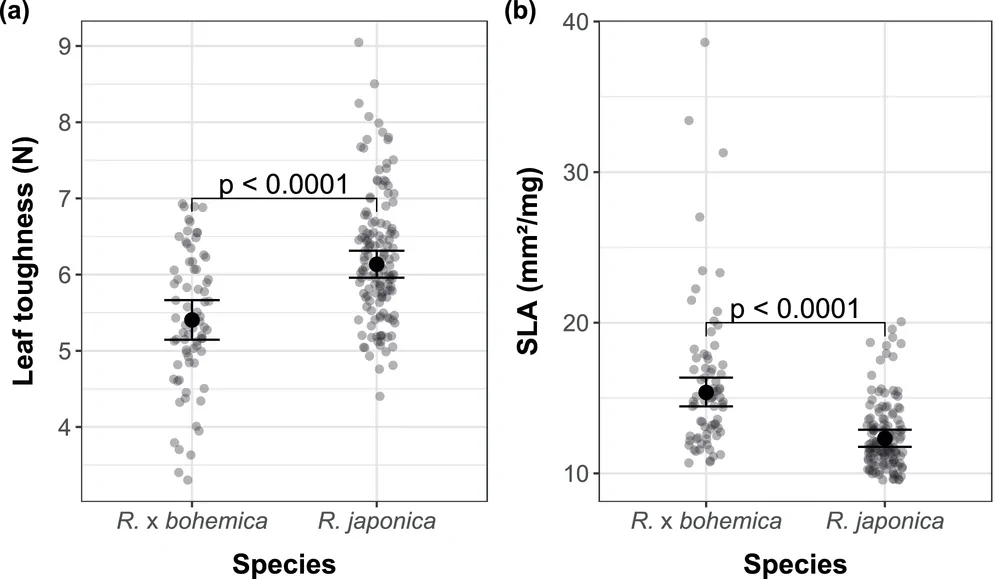
(a) Leaf toughness (Newtons [N]) and (b) SLA (mm^2^/mg) of *R*. × *bohemica* and *R. japonica* . Semi‐transparent points are raw data, while larger points and error bars depict model‐predicted estimated marginal means and 95% CI from our best LMMs of leaf toughness and SLA, respectively (Table 1).

Variation in herbivore performance (i.e., survival, development time) and leaf traits (i.e., leaf toughness, SLA) was best explained by including species (i.e., *R*. × *bohemica* vs. *R. japonica* ) in our models (Table 1). Survival of *S. exigua* to the pupal stage was significantly lower (29% difference; *z* = 3.53, *p* < 0.001), and development time significantly increased (5.3 days to pupal stage, 27% difference; *t*_22.5_ = −5.03, *p* < 0.001; 5.6 days to adult stage, 21% difference; *t*_16.9_ = −3.02, *p* < 0.01), on *R. japonica* compared to *R*. × *bohemica* (Figures 1 and 2; Table 1; Table S21). Leaves were significantly tougher (13% [0.73 N] difference; *t*_63.3_ = −4.66, *p* < 0.001) and lower in SLA (20% [3 mm^2^/mg] difference; *t*_65.9_ = 5.89, *p* < 0.001) for *R. japonica* compared to *R*. × *bohemica* (Figure 3; Table 1; Table S21).

**FIGURE 4:**
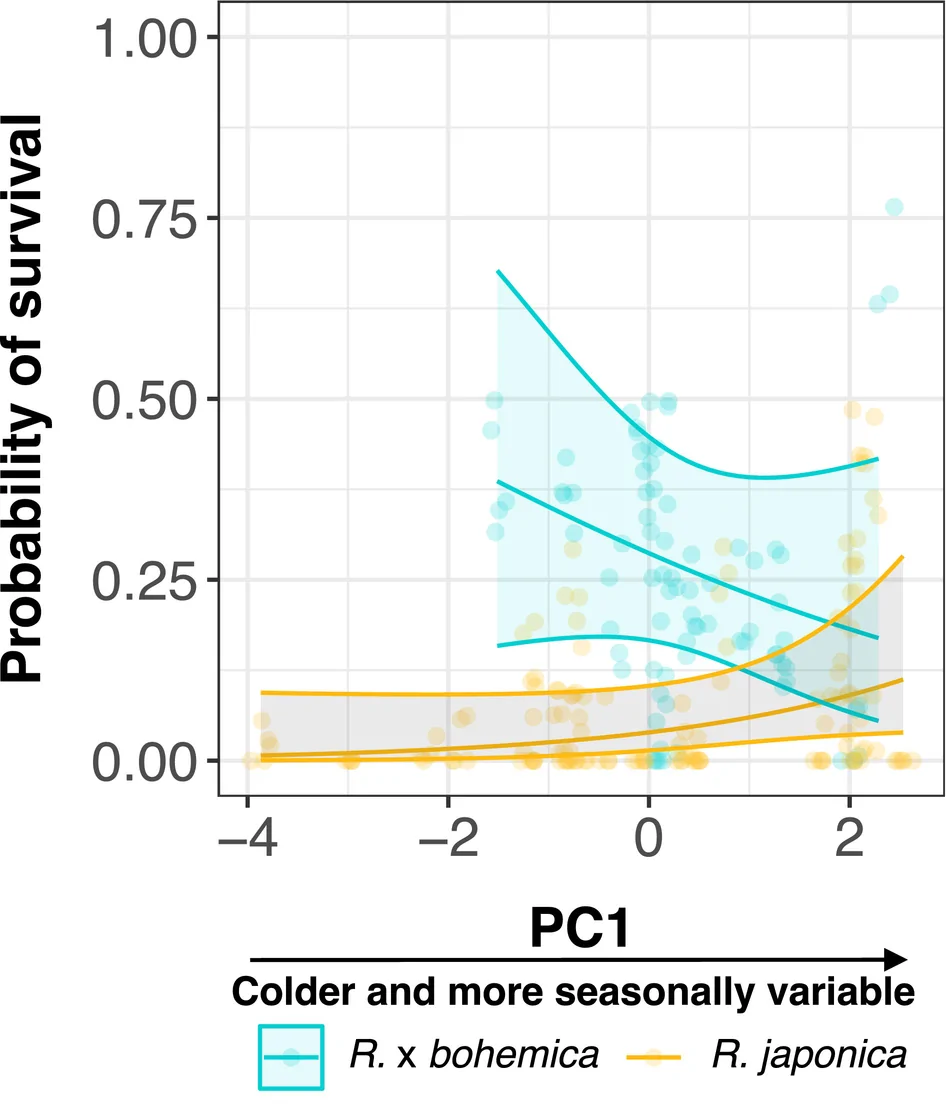
Probability of *S. exigua* survival to adult when fed *R*. × *bohemica* (blue) or *R. japonica* (yellow) as a function of variation in climate conditions at original collection locations. As values of PC1 increase, climates at collection locations are colder and have larger annual temperature ranges. Semi‐transparent points are raw data, while the line and standard error ribbon depict model‐predicted estimated marginal means and 95% CI from our best GLMM of survival to adult (Table 1).

**FIGURE 5:**
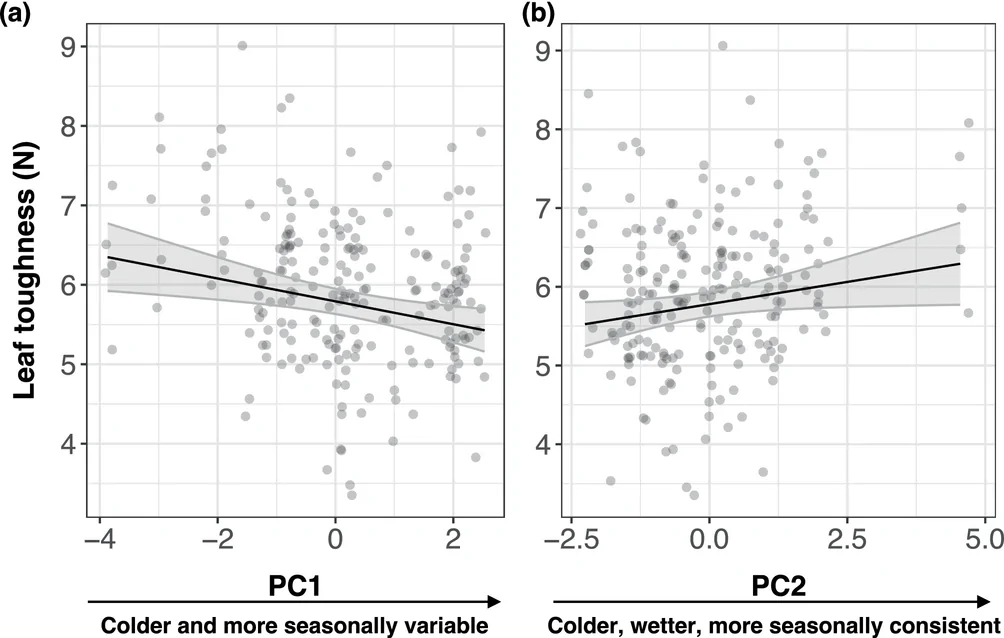
Leaf toughness (Newtons [N]) of *Reynoutria* spp. as a function of variation in climate conditions at original collection locations. (a) As values of PC1 increase, climates at collection locations are colder and have larger annual temperature ranges. (b) As values of PC2 increase, climates at collection locations are colder, wetter, and precipitation is more seasonally consistent. Semi‐transparent points are raw data, while the line and standard error ribbon depict model‐predicted estimated marginal means and 95% CI from our best LMM of leaf toughness (Table 1).

Variation in herbivore performance (i.e., survival, adult weight) and leaf toughness was also better explained by models that included at least one of the three climate PCs rather than latitude at original collection locations (Table 1). Survival of *S. exigua* to the adult stage was significantly higher on *R*. × *bohemica* compared to *R. japonica* in warmer and more seasonally consistent climates; however, survival on both species became increasingly similar as survival increased on *R. japonica* , and decreased on *R*. × *bohemica*, as climates at collection locations became colder and more seasonally variable (species × PC1: 𝜒^2^(1) = 3.95, *p* = 0.047) (Figure 4; Table 1; Table S21). Leaf toughness also increased as climates at collection locations became “wetter” and more seasonally consistent (Figure 5; Table 1; Table S21). When assessing the effects of climate and latitude in the same models, all but two of the best overall models remained the same: the two models explaining variation in *S. exigua* survival to the adult stage and leaf toughness included latitude instead of PC1; all other variables and their interactions remained unchanged (Tables S14 and S19).

## 4 Discussion

Our results implicate the ecological importance of species‐level variation, potentially mediated by hybridization, polyploidy, and other linked evolutionary processes, in shaping genetically based resistance to herbivory in two very closely related non‐native species. Interspecific differences were more important in explaining heritable variation in resistance than climate or latitude at collection locations across the introduced range in North America. This pattern was consistent for measures of herbivore performance as well as for key functional leaf traits known to affect herbivory. Furthermore, climate generally explained more variation than non‐climatic correlates of latitude, but similar clinal patterns remained significant regardless of whether climate and latitude were compared in the same or in different models. Thus, by explicitly testing effects of both, we confirm that latitude is an acceptable proxy of how climate mediates selection within the knotweed system, but may also capture other unexplored environmental and biotic gradients that vary across geographic scales in other systems. Our findings are especially notable, as *R. japonica* is often described as a highly successful invader in North America despite originating from a single dominant genotype or small founding population (Burns et al. 2025; Gaskin et al. 2014; Groeneveld et al. 2014). Yet, recent work indicates greater genetic diversity within the knotweed complex in its introduced range than previously reported (Tippery et al. 2021; VanWallendael et al. 2021), and we show that this variation can drive selection in key functional leaf traits that affect herbivore performance.

We found lower levels of resistance to herbivory in the hybrid polyploid *R*. × *bohemica* compared to its parent *R. japonica* . Hybridization may produce either intermediate phenotypes or novel recombinant traits that diverge from parental species following subsequent selection (Fritz et al. 1999; Rieseberg et al. 2007). Changes in ploidy associated with hybridization can also increase cell and genome size at higher ploidy levels (Müntzing 1936; Stebbins 1971), which may in turn contribute to genetically based variation in structural leaf traits that shape patterns of herbivory. Resistance can vary with herbivore identity, plant taxa, and plant genotype (Halverson et al. 2008; Nuismer and Thompson 2001), but may also differ among functional traits, that is polyploids often have increased concentrations of secondary metabolites (e.g., polyphenolics and alkaloids) that reduce herbivore performance, but may also have higher leaf water and dry matter content that may decrease resistance to herbivory (DeRose et al. 2022; Meyerson et al. 2016; Stutz et al. 2016). Follow‐up experiments incorporating both multiple herbivore species (including specialists from the native range) and resistance traits may help disentangle the specific plant traits underlying variation in herbivore performance and assess whether generalizable patterns of resistance emerge across plant and herbivore species that influence invasion success.

Common gardens are a powerful tool for investigating local adaptation to climate or other factors that may also vary with latitude. By growing populations under standardized conditions (i.e., same potting medium, watering regime, and location), we can minimize the contribution of environmentally induced variation and more clearly distinguish genetically based phenotypic differences (Colautti and Lau 2015). Our study therefore sets the stage for future common garden experiments across both native and introduced ranges to disentangle the competing selective forces shaping variation in different herbivore resistance or tolerance strategies. Given significant enemy release in knotweed's introduced range, selection by herbivores on resistance traits is likely weak, but the absence of specialists and reduced herbivory by generalists may also reshape the evolution of these traits and their contribution to variation in invasion success (Blossey and Nötzold 1995; Callaway et al. 2022; Sun et al. 2023). For example, Sun et al. (2023) showed that constitutive defenses in introduced populations were positively correlated, and induced defenses negatively correlated, with intensity of herbivory. Constitutive and induced defenses may also respond differently to environmental variation (Moreira et al. 2014): different resistance traits vary in their ecological costs and benefits (Karban 2011) and are differentially expressed depending on abiotic and biotic conditions (Sampedro et al. 2011). However, without native‐range comparisons, and explicit assessments of induced versus constitutive defenses, we cannot determine whether the observed differences—while consistent with evolutionary differentiation between species and cytotypes—evolved prior to or after introduction to a new range, and whether they extend to other types of defense strategies (Sun et al. 2023; van Kleunen et al. 2010).

Given significant enemy release in the introduced range, knotweed populations may now be shaped less by herbivore pressure and more by strong selection to optimize adaptation to local climate conditions. We explicitly evaluated both climatic and latitudinal effects in our study to help tease apart variation in leaf traits and herbivore performance explained by climate versus latitude. By explicitly partitioning climatic versus non‐climatic factors across both competing models and within the same models, we show that latitude was an acceptable proxy for climatic variation within the knotweed system. However, latitude may also capture other biotic and abiotic selection pressures not directly measured in this study that may drive trait selection in other systems. For example, unexplored environmental variables, such as daily and seasonal photoperiods, often vary predictably with latitude (Jackson 2009), yet are often difficult to disentangle from concurrent effects of temperature along latitudinal clines (De Frenne et al. 2013). Furthermore, latitudinal and altitudinal gradients differ in environmental factors that can produce divergent patterns of genetic differentiation and local adaptation, emphasizing the need for integrated approaches rather than relying on a single proxy for multiple environmental gradients (De Frenne et al. 2013). More broadly, our findings showcase an important method for providing important insights in other systems about why empirical tests of prominent theories of clinal variation in plant defenses, such as the latitudinal herbivory‐defense hypothesis (Coley and Aide 1991) and resource availability hypothesis (Coley et al. 1985), often yield conflicting predictions when underlying environmental gradients are not disentangled.

Overall, our findings emphasize the critical role of interspecific differences, even for extremely closely related species linked by interacting genetic and evolutionary processes, in shaping heritable variation in resistance to herbivory across novel environments. Exploring underlying factors that may explain predictable geographical variation in herbivory and resistance traits has gained momentum in recent years (Anstett et al. 2016; Hahn et al. 2019; Rasmann et al. 2014; The Herbivory Variability Network 2023). Yet, accumulating conflicting evidence for the core theories tested within these studies reflects the complex suite of selective forces shaping geographical variation in plant defenses and the difficulty in teasing apart the impacts of these different pressures (Anstett et al. 2016; Stevens et al. 2016; Kooyers et al. 2017; Moreira et al. 2018). How intra‐ and interspecific genetic variation interacts with these environmental factors to concurrently shape trait expression, especially in ways that influence invasion success, remains unclear and may help reconcile discrepancies in observed patterns of herbivory and defense across diverse taxa (Meyerson et al. 2016). Our approach—assessing how heritable variation in herbivore performance and key functional traits can be explained by climate, latitude, or interspecific genetic differences across diverse biotic and abiotic environments—could therefore serve as a model for uncovering key ecological and evolutionary patterns in other systems.

## Author Contributions

**Katie Lee:** conceptualization (equal), formal analysis (lead), investigation (lead), methodology (equal), writing – original draft (lead), writing – review and editing (equal). **Stacy B. Endriss:** conceptualization (equal), formal analysis (supporting), investigation (supporting), methodology (equal), writing – review and editing (equal). **Bernd Blossey:** conceptualization (equal), funding acquisition (lead), methodology (equal), writing – review and editing (equal).

## Funding

This work was supported by the New York Department of Environmental Conservation through the New York Invasive Species Research Institute.

## Conflicts of Interest

The authors declare no conflicts of interest.

## Supporting information


**Figure S1:** Map of original collection locations for rhizomes of *R. japonica* (RJ, blue circles), *R*. × *bohemica* (RB, red circles), and both knotweed species (RJ/RB, green circles). Locations are referenced in Table S1.
**Figure S2:** (a) Scaled principal component analysis (PCA) of climate data (WorldClim bioclimatic variables) across knotweed's introduced North American range. Bioclimatic variables are labeled as follows: bio2 = mean diurnal range (mean of monthly [maximum temperature—minimum temperature]), bio7 = temperature annual range (maximum temperature of warmest month—minimum temperature of coldest month), bio8 = mean temperature of wettest quarter, bio9 = mean temperature of driest quarter, bio10 = mean temperature of warmest quarter, bio12 = annual precipitation, and bio15 = precipitation seasonality (coefficient of variation). (b) Variable loadings onto each PC, with negative loadings in red and positive loadings in blue. PC1 explained 37.4%, PC2 28.3%, and PC3 16.7% of the overall variation in climate. Collectively, the three PCs explained 82.3% of the total variation in climatic data. PC1 and PC2 were both attributed mainly to temperature and precipitation. As values of PC1 increase, climates at collection locations are colder (bio10, bio9) and have larger annual temperature ranges (bio7). As values of PC2 increase, climates at collection locations are colder (bio8), wetter (bio12), and precipitation is more seasonally consistent (bio15). In contrast, PC3 was attributed mainly to climate seasonality: as values of PC3 increase, monthly temperature ranges become smaller (bio2) and precipitation is more seasonally consistent (bio15).
**Table S1:** Collection locations (state, latitude, longitude) and taxa identification (based on ploidy [ *R. japonica* = 8*x*, *R*. × *bohemica* = 6*x*]) of each plant represented in our common garden. The individual IDs and taxa of plants used in the herbivore performance study are bolded. State two letter designations are: GA (Georgia), NC (North Carolina), VA (Virginia), MD (Maryland), PA (Pennsylvania), NJ (New Jersey), NY (New York), MA (Massachusetts), VT (Vermont), NH (New Hampshire), ME (Maine). Four collection locations were excluded from analyses as they contained *R. sachalinensis* (4*x*) plants.
**Table S2:** Summary of the best models (defined as models with the lowest AICc values) that include latitude fit to data of survival of *S. exigua* to the pupal and adult stages, adult weight of *S. exigua* , and leaf toughness (i.e., variables for which the best overall models included climate; see Table 1). Models of knotweed traits included collection location ID, or collection location ID and shelf for models of herbivore performance, as random intercepts. Significant main and interactive effects (*p* < 0.05) in each model are bolded, and their marginal means or trends calculated using *emmeans* and *emtrends* as discussed in the model interpretation column.
**Table S3:** We employed the R function *dredge* to select the best of our generalized linear mixed models (i.e., model with the lowest AICc value) for assessing effects of climate (PC1, PC2, PC3), latitude, and interspecific differences (species) on survival of *S. exigua* to the pupal stage. We fit all models with random intercepts of collection location ID and shelf. *K* refers to the number of estimable parameters in a model. ΔAICc is calculated by subtracting the AICc value of the best model from the AICc of the given model. Akaike weight (*w*_i_) is the relative likelihood that a given model is the best of a set of models. Only the top models (defined as models with ΔAICc < 2 from the best model; Burnham and Anderson 2002) are presented (see code for full model set).
**Table S4:** We employed the R function *dredge* to select the best of our generalized linear mixed models (i.e., model with the lowest AICc value) for assessing effects of climate (PC1, PC2, PC3), latitude, and interspecific differences (species) on survival of *S. exigua* to the adult stage. We fit all models with random intercepts of collection location ID and shelf. *K* refers to the number of estimable parameters in a model. ΔAICc is calculated by subtracting the AICc value of the best model from the AICc of the given model. Akaike weight (*w*_i_) is the relative likelihood that a given model is the best of a set of models. Only the top models (defined as models with ΔAICc < 2 from the best model; Burnham and Anderson 2002) are presented (see code for full model set).
**Table S5:** We employed the R function *dredge* to select the best of our linear mixed models (i.e., model with the lowest AICc value) for assessing effects of climate (PC1, PC2, PC3), latitude, and interspecific differences (species) on pupal weight of *S. exigua* . We fit all models with random intercepts of collection location ID and shelf. *K* refers to the number of estimable parameters in a model. ΔAICc is calculated by subtracting the AICc value of the best model from the AICc of the given model. Akaike weight (*w*_i_) is the relative likelihood that a given model is the best of a set of models. Only the top models (defined as models with ΔAICc < 2 from the best model; Burnham and Anderson 2002) are presented (see code for full model set).
**Table S6:** We employed the R function *dredge* to select the best of our linear mixed models (i.e., model with the lowest AICc value) for assessing effects of climate (PC1, PC2, PC3), latitude, and interspecific differences (species) on adult weight of *S. exigua* . We fit all models with random intercepts of collection location ID and shelf. *K* refers to the number of estimable parameters in a model. ΔAICc is calculated by subtracting the AICc value of the best model from the AICc of the given model. Akaike weight (*w*_i_) is the relative likelihood that a given model is the best of a set of models. Only the top models (defined as models with ΔAICc < 2 from the best model; Burnham and Anderson 2002) are presented (see code for full model set).
**Table S7:** We employed the R function *dredge* to select the best of our linear mixed models (i.e., model with the lowest AICc value) for assessing effects of climate (PC1, PC2, PC3), latitude, and interspecific differences (species) on development time of *S. exigua* to the pupal stage. We fit all models with random intercepts of collection location ID and shelf. *K* refers to the number of estimable parameters in a model. ΔAICc is calculated by subtracting the AICc value of the best model from the AICc of the given model. Akaike weight (*w*_i_) is the relative likelihood that a given model is the best of a set of models. Only the top models (defined as models with ΔAICc < 2 from the best model; Burnham and Anderson 2002) are presented (see code for full model set).
**Table S8:** We employed the R function *dredge* to select the best of our linear mixed models (i.e., model with the lowest AICc value) for assessing effects of climate (PC1, PC2, PC3), latitude, and interspecific differences (species) on development time of *S. exigua* to the adult stage. We fit all models with random intercepts of collection location ID and shelf. *K* refers to the number of estimable parameters in a model. ΔAICc is calculated by subtracting the AICc value of the best model from the AICc of the given model. Akaike weight (*w*_i_) is the relative likelihood that a given model is the best of a set of models. Only the top models (defined as models with ΔAICc < 2 from the best model; Burnham and Anderson 2002) are presented (see code for full model set).
**Table S9:** We employed the R function *dredge* to select the best of our linear mixed models (i.e., model with the lowest AICc value) for assessing effects of climate (PC1, PC2, PC3), latitude, and interspecific differences (species) on leaf toughness. We fit all models with a random intercept of collection location ID. *K* refers to the number of estimable parameters in a model. ΔAICc is calculated by subtracting the AICc value of the best model from the AICc of the given model. Akaike weight (*w*_i_) is the relative likelihood that a given model is the best of a set of models. Only the top models (defined as models with ΔAICc < 2 from the best model; Burnham and Anderson 2002) are presented (see code for full model set).
**Table S10:** We employed the R function *dredge* to select the best of our linear mixed models (i.e., model with the lowest AICc value) for assessing effects of climate (PC1, PC2, PC3), latitude, and interspecific differences (species) on SLA. We fit all models with a random intercept of collection location ID. *K* refers to the number of estimable parameters in a model. ΔAICc is calculated by subtracting the AICc value of the best model from the AICc of the given model. Akaike weight (*w*_i_) is the relative likelihood that a given model is the best of a set of models. Only the top models (defined as models with ΔAICc < 2 from the best model; Burnham and Anderson 2002) are presented (see code for full model set).
**Table S11:** Descriptive statistics (mean, standard deviation, minimum, maximum) for leaf toughness (tough) and SLA among plants within collection locations. Location coordinates (latitude, longitude) and number of plants sampled per location (N) are referenced in Table S1.
**Table S12:** Bioclimatic variable loadings on PC1, PC2, and PC3 (see Figure S2).
**Table S13:** We employed the R function *dredge* to select the best of our generalized linear mixed models (i.e., model with the lowest AICc value) for assessing effects of climate (PC2 and PC3), latitude (instead of climate PC1), and interspecific differences (species) on survival of *S. exigua* to the pupal stage. We fit all models with random intercepts of collection location ID and shelf. *K* refers to the number of estimable parameters in a model. ΔAICc is calculated by subtracting the AICc value of the best model from the AICc of the given model. Akaike weight (*w*_i_) is the relative likelihood that a given model is the best of a set of models. Only the top models (defined as models with ΔAICc < 2 from the best model; Burnham and Anderson 2002) are presented (see code for full model set). Significant main and interactive effects (*p* < 0.05) in the best model are bolded.
**Table S14:** We employed the R function *dredge* to select the best of our generalized linear mixed models (i.e., model with the lowest AICc value) for assessing effects of climate (PC2 and PC3), latitude (instead of climate PC1), and interspecific differences (species) on survival of *S. exigua* to the adult stage. We fit all models with random intercepts of collection location ID and shelf. *K* refers to the number of estimable parameters in a model. ΔAICc is calculated by subtracting the AICc value of the best model from the AICc of the given model. Akaike weight (*w*_i_) is the relative likelihood that a given model is the best of a set of models. Only the top models (defined as models with ΔAICc < 2 from the best model; Burnham and Anderson 2002) are presented (see code for full model set). Significant main and interactive effects (*p* < 0.05) in the best model are bolded.
**Table S15:** We employed the R function *dredge* to select the best of our linear mixed models (i.e., model with the lowest AICc value) for assessing effects of climate (PC2 and PC3), latitude (instead of climate PC1), and interspecific differences (species) on pupal weight of *S. exigua* . We fit all models with random intercepts of collection location ID and shelf. *K* refers to the number of estimable parameters in a model. ΔAICc is calculated by subtracting the AICc value of the best model from the AICc of the given model. Akaike weight (*w*_i_) is the relative likelihood that a given model is the best of a set of models. Only the top models (defined as models with ΔAICc < 2 from the best model; Burnham and Anderson 2002) are presented (see code for full model set). Significant main and interactive effects (*p* < 0.05) in the best model are bolded.
**Table S16:** We employed the R function *dredge* to select the best of our linear mixed models (i.e., model with the lowest AICc value) for assessing effects of climate (PC2 and PC3), latitude (instead of climate PC1), and interspecific differences (species) on adult weight of *S. exigua* . We fit all models with random intercepts of collection location ID and shelf. *K* refers to the number of estimable parameters in a model. ΔAICc is calculated by subtracting the AICc value of the best model from the AICc of the given model. Akaike weight (*w*_i_) is the relative likelihood that a given model is the best of a set of models. Only the top models (defined as models with ΔAICc < 2 from the best model; Burnham and Anderson 2002) are presented (see code for full model set). Significant main and interactive effects (*p* < 0.05) in the best model are bolded.
**Table S17:** We employed the R function *dredge* to select the best of our linear mixed models (i.e., model with the lowest AICc value) for assessing effects of climate (PC2 and PC3), latitude (instead of climate PC1), and interspecific differences (species) on development time of *S. exigua* to the pupal stage. We fit all models with random intercepts of collection location ID and shelf. *K* refers to the number of estimable parameters in a model. ΔAICc is calculated by subtracting the AICc value of the best model from the AICc of the given model. Akaike weight (*w*_i_) is the relative likelihood that a given model is the best of a set of models. Only the top models (defined as models with ΔAICc < 2 from the best model; Burnham and Anderson 2002) are presented (see code for full model set). Significant main and interactive effects (*p* < 0.05) in the best model are bolded.
**Table S18:** We employed the R function *dredge* to select the best of our linear mixed models (i.e., model with the lowest AICc value) for assessing effects of climate (PC2 and PC3), latitude (instead of climate PC1), and interspecific differences (species) on development time of *S. exigua* to the adult stage. We fit all models with random intercepts of collection location ID and shelf. *K* refers to the number of estimable parameters in a model. ΔAICc is calculated by subtracting the AICc value of the best model from the AICc of the given model. Akaike weight (*w*_i_) is the relative likelihood that a given model is the best of a set of models. Only the top models (defined as models with ΔAICc < 2 from the best model; Burnham and Anderson 2002) are presented (see code for full model set). Significant main and interactive effects (*p* < 0.05) in the best model are bolded.
**Table S19:** We employed the R function *dredge* to select the best of our linear mixed models (i.e., model with the lowest AICc value) for assessing effects of climate (PC2 and PC3), latitude (instead of climate PC1), and interspecific differences (species) on leaf toughness. We fit all models with a random intercept of collection location ID. *K* refers to the number of estimable parameters in a model. ΔAICc is calculated by subtracting the AICc value of the best model from the AICc of the given model. Akaike weight (*w*_i_) is the relative likelihood that a given model is the best of a set of models. Only the top models (defined as models with ΔAICc < 2 from the best model; Burnham and Anderson 2002) are presented (see code for full model set). Significant main and interactive effects (*p* < 0.05) in the best model are bolded.
**Table S20:** We employed the R function *dredge* to select the best of our linear mixed models (i.e., model with the lowest AICc value) for assessing effects of climate (PC2 and PC3), latitude (instead of climate PC1), and interspecific differences (species) on SLA. We fit all models with a random intercept of collection location ID. *K* refers to the number of estimable parameters in a model. ΔAICc is calculated by subtracting the AICc value of the best model from the AICc of the given model. Akaike weight (*w*_i_) is the relative likelihood that a given model is the best of a set of models. Only the top models (defined as models with ΔAICc < 2 from the best model; Burnham and Anderson 2002) are presented (see code for full model set). Significant main and interactive effects (*p* < 0.05) in the best model are bolded.
**Table S21:** Pairwise comparisons for significant main and interactive effects in the best models fit to data of herbivore performance and knotweed leaf traits. Significant main and interactive effects (*p* < 0.05) are bolded. An odds ratio greater than one indicates that the first group has a higher likelihood of surviving than the second group. A positive mean difference or ratio (SLA) greater than one indicates that the first group has a higher estimate than the second group. PC1, PC2, and PC3 represent the three linear principal components (PCs) for climate.

## Data Availability

Code and data that support the findings of this research are available from the Figshare Digital Repository: https://figshare.com/s/f8ef7c1217f0c85770cf (Lee et al. 2026).
