# Supplementary material for: Species Identity Drives Geographic Variation in Leaf Traits and Palatability to a Generalist Insect Herbivore of a Common Invader
Source: Ecol Evol. 2026 Sep 23;16(9):e74387. doi: 10.1002/ece3.74387 (PMC13601769; doi:10.1002/ece3.74387)
Supplement: Supplementary file 1 [file ECE3-16-e74387-s001.docx]

**Supporting Information**

**Species identity drives geographic variation in leaf traits and palatability to a generalist insect herbivore of a common invader**

**Figure S1.** Map of original collection locations for rhizomes of *R. japonica* (RJ, blue circles), *R.* × *bohemica* (RB, red circles), and both knotweed species (RJ/RB, green circles). Locations are referenced in Table S1.

**(a)	(b)**

**Figure S2.** (a) Scaled principal component analysis (PCA) of climate data (WorldClim bioclimatic variables) across knotweed’s introduced North American range. Bioclimatic variables are labeled as follows: bio2 = mean diurnal range (mean of monthly [maximum temperature - minimum temperature]), bio7 = temperature annual range (maximum temperature of warmest month - minimum temperature of coldest month), bio8 = mean temperature of wettest quarter, bio9 = mean temperature of driest quarter, bio10 = mean temperature of warmest quarter, bio12 = annual precipitation, and bio15 = precipitation seasonality (coefficient of variation). (b) Variable loadings onto each PC, with negative loadings in red and positive loadings in blue. PC1 explained 37.4%, PC2 28.3%, and PC3 16.7% of the overall variation in climate. Collectively, the three PCs explained 82.3% of the total variation in climatic data. PC1 and PC2 were both attributed mainly to temperature and precipitation. As values of PC1 increase, climates at collection locations are colder (bio10, bio9) and have larger annual temperature ranges (bio7). As values of PC2 increase, climates at collection locations are colder (bio8), wetter (bio12), and precipitation is more seasonally consistent (bio15). In contrast, PC3 was attributed mainly to climate seasonality: as values of PC3 increase, monthly temperature ranges become smaller (bio2) and precipitation is more seasonally consistent (bio15).

**Table S1.** Collection locations (state, latitude, longitude) and taxa identification (based on ploidy [*R. japonica* = 8x, *R.* × *bohemica* = 6x]) of each plant represented in our common garden. The individual IDs and taxa of plants used in the herbivore performance study are bolded. State two letter designations are: GA (Georgia), NC (North Carolina), VA (Virginia), MD (Maryland), PA (Pennsylvania), NJ (New Jersey), NY (New York), MA (Massachusetts), VT (Vermont), NH (New Hampshire), ME (Maine). Four collection locations were excluded from analyses as they contained *R. sachalinensis* (4x) plants.

| **Individual** | **State** | **Taxa** | **Latitude** | **Longitude** |
| --- | --- | --- | --- | --- |
| **1** | GA | ***R. japonica*** | 34.24369 | -83.45888 |
| 2 |  | *R. japonica* |  |  |
| 3 |  | *R. japonica* |  |  |
| 4 |  | *R. japonica* |  |  |
| 5 |  | *R. japonica* |  |  |
| 1 | NC | *R. japonica* | 35.10431 | -83.09636 |
| **2** |  | ***R. japonica*** |  |  |
| 3 |  | *R. japonica* |  |  |
| 4 |  | *R. japonica* |  |  |
| 5 |  | *R. japonica* |  |  |
| 1 | NC | *R. japonica* | 35.46315 | -83.00964 |
| 2 |  | *R. japonica* |  |  |
| 3 |  | *R. japonica* |  |  |
| **4** |  | ***R. japonica*** |  |  |
| 5 |  | *R. japonica* |  |  |
| 1 | NC | *R. japonica* | 35.73982 | -82.67967 |
| 2 |  | *R. japonica* |  |  |
| **3** |  | ***R. japonica*** |  |  |
| 4 |  | *R. japonica* |  |  |
| 5 |  | *R. japonica* |  |  |
| 1 | NC | *R. japonica* | 36.07876 | -82.34724 |
| **2** |  | ***R.*** × ***bohemica*** |  |  |
| 3 |  | *R.* × *bohemica* |  |  |
| 4 |  | *R.* × *bohemica* |  |  |
| 5 |  | *R.* × *bohemica* |  |  |
| 1 | NC | *R. japonica* | 36.21330 | -81.77665 |
| 2 |  | *R. japonica* |  |  |
| **3** |  | ***R. japonica*** |  |  |
| 4 |  | *R. japonica* |  |  |
| 5 |  | *R. japonica* |  |  |
| **1** | NC | ***R. japonica*** | 36.37824 | -81.37687 |
| 2 |  | *R. japonica* |  |  |
| 3 |  | *R. japonica* |  |  |
| 5 |  | *R. japonica* |  |  |
| 1 | VA | *R. japonica* | 36.64657 | -80.91819 |
| 2 |  | *R. japonica* |  |  |
| 3 |  | *R. japonica* |  |  |
| **4** |  | ***R. japonica*** |  |  |
| 5 |  | *R. japonica* |  |  |
| 1 | VA | *R. japonica* | 37.13998 | -80.54223 |
| 2 |  | *R. japonica* |  |  |
| 3 |  | *R. japonica* |  |  |
| **4** |  | ***R. japonica*** |  |  |
| 5 |  | *R. japonica* |  |  |
| 1 | VA | *R. japonica* | 37.27712 | -80.05605 |
| **2** |  | ***R. japonica*** |  |  |
| 3A |  | *R. japonica* |  |  |
| 3B |  | *R. japonica* |  |  |
| 4 |  | *R. japonica* |  |  |
| 5 |  | *R. japonica* |  |  |
| 2 | VA | *R. japonica* | 37.36660 | -79.40806 |
| **4** |  | ***R. japonica*** |  |  |
| **1** | VA | ***R. japonica*** | 37.42545 | -79.15961 |
| 2 |  | *R. japonica* |  |  |
| 3 |  | *R. japonica* |  |  |
| 4 |  | *R. japonica* |  |  |
| 5 |  | *R. japonica* |  |  |
| **1** | VA | ***R.*** × ***bohemica*** | 38.06942 | -78.88527 |
| 2 |  | *R.* × *bohemica* |  |  |
| 3 |  | *R.* × *bohemica* |  |  |
| 4 |  | *R.* × *bohemica* |  |  |
| 5 |  | *R.* × *bohemica* |  |  |
| 1 | VA | *R. japonica* | 38.45577 | -78.59494 |
| 2 |  | *R. japonica* |  |  |
| **4** |  | ***R. japonica*** |  |  |
| 5 |  | *R. japonica* |  |  |
| 1 | VA | *R. japonica* | 38.64652 | -78.53521 |
| 2 |  | *R. japonica* |  |  |
| **3** |  | ***R. japonica*** |  |  |
| 4 |  | *R. japonica* |  |  |
| 5 |  | *R. japonica* |  |  |
| 1 | VA | *R. japonica* | 39.08063 | -78.08574 |
| **2** |  | ***R. japonica*** |  |  |
| 3 |  | *R. japonica* |  |  |
| 4 |  | *R. japonica* |  |  |
| 5 |  | *R.* × *bohemica* |  |  |
| 1 | MD | *R. japonica* | 39.27235 | -77.54727 |
| 3 |  | *R.* × *bohemica* |  |  |
| **4** |  | ***R.*** × ***bohemica*** |  |  |
| 5 |  | *R.* × *bohemica* |  |  |
| 1 | MD | *R. japonica* | 39.61071 | -76.68270 |
| 2 |  | *R. japonica* |  |  |
| 3 |  | *R. japonica* |  |  |
| 4 |  | *R. japonica* |  |  |
| **5** |  | ***R. japonica*** |  |  |
| **1** | PA | ***R.*** × ***bohemica*** | 39.79606 | -76.73519 |
| 3 |  | *R.* × *bohemica* |  |  |
| 4 |  | *R.* × *bohemica* |  |  |
| 1 | PA | *R.* × *bohemica* | 40.09314 | -76.67698 |
| 2 |  | *R.* × *bohemica* |  |  |
| **3** |  | ***R.*** × ***bohemica*** |  |  |
| 4 |  | *R.* × *bohemica* |  |  |
| 5 |  | *R.* × *bohemica* |  |  |
| 1 | PA | *R.* × *bohemica* | 40.16614 | -76.29577 |
| 2 |  | *R.* × *bohemica* |  |  |
| 3 |  | *R.* × *bohemica* |  |  |
| **4** |  | ***R.*** × ***bohemica*** |  |  |
| 5 |  | *R.* × *bohemica* |  |  |
| 1 | PA | *R.* × *bohemica* | 40.28092 | -75.94827 |
| 2 |  | *R.* × *bohemica* |  |  |
| **3** |  | ***R.*** × ***bohemica*** |  |  |
| 4 |  | *R.* × *bohemica* |  |  |
| 5 |  | *R.* × *bohemica* |  |  |
| **1** | PA | ***R.*** × ***bohemica*** | 40.34566 | -75.48387 |
| 2 |  | *R.* × *bohemica* |  |  |
| 3 |  | *R.* × *bohemica* |  |  |
| 4 |  | *R.* × *bohemica* |  |  |
| 5 |  | *R.* × *bohemica* |  |  |
| **1** | PA | ***R.*** × ***bohemica*** | 40.63584 | -75.39360 |
| 2 |  | *R.* × *bohemica* |  |  |
| 3 |  | *R.* × *bohemica* |  |  |
| 4 |  | *R.* × *bohemica* |  |  |
| 5 |  | *R.* × *bohemica* |  |  |
| 1 | NJ | *R.* × *bohemica* | 40.73400 | -75.08229 |
| 2 |  | *R.* × *bohemica* |  |  |
| 3 |  | *R.* × *bohemica* |  |  |
| 4 |  | *R.* × *bohemica* |  |  |
| **5** |  | ***R.*** × ***bohemica*** |  |  |
| **1** | NJ | ***R.*** × ***bohemica*** | 40.80224 | -74.69688 |
| 2 |  | *R. japonica* |  |  |
| 3 |  | *R.* × *bohemica* |  |  |
| 4 |  | *R.* × *bohemica* |  |  |
| 5 |  | *R.* × *bohemica* |  |  |
| 1 | NJ | *R. japonica* | 41.01127 | -74.35119 |
| 2 |  | *R. japonica* |  |  |
| 3 |  | *R. japonica* |  |  |
| 4 |  | *R. japonica* |  |  |
| **5** |  | ***R. japonica*** |  |  |
| 1 | NY | *R.* × *bohemica* | 41.20022 | -74.19431 |
| 2 |  | *R.* × *bohemica* |  |  |
| 3 |  | *R.* × *bohemica* |  |  |
| **4** |  | ***R.*** × ***bohemica*** |  |  |
| 5 |  | *R.* × *bohemica* |  |  |
| **1** | NY | ***R. japonica*** | 41.41457 | -73.95776 |
| 2 |  | *R. japonica* |  |  |
| 3 |  | *R. japonica* |  |  |
| 4 |  | *R. japonica* |  |  |
| 5 |  | *R. japonica* |  |  |
| 1 | NY | *R. japonica* | 41.76539 | -73.86471 |
| 2 |  | *R. japonica* |  |  |
| 3 |  | *R. japonica* |  |  |
| 4 |  | *R. japonica* |  |  |
| **5** |  | ***R. japonica*** |  |  |
| 1 | NY | *R.* × *bohemica* | 41.95216 | -73.51009 |
| 2 |  | *R.* × *bohemica* |  |  |
| **3** |  | ***R.*** × ***bohemica*** |  |  |
| 5 |  | *R.* × *bohemica* |  |  |
| 1 | MA | *R.* × *bohemica* | 42.28011 | -73.29808 |
| 2 |  | *R.* × *bohemica* |  |  |
| **4** |  | ***R.*** × ***bohemica*** |  |  |
| 5 |  | *R. japonica* |  |  |
| 1 | MA | *R.* × *bohemica* | 42.38676 | -72.53005 |
| 2 |  | *R.* × *bohemica* |  |  |
| **3** |  | ***R.*** × ***bohemica*** |  |  |
| 4 |  | *R.* × *bohemica* |  |  |
| 5 |  | *R.* × *bohemica* |  |  |
| **1** | MA | ***R. japonica*** | 42.63896 | -72.91260 |
| 2 |  | *R. japonica* |  |  |
| 3 |  | *R. japonica* |  |  |
| 4 |  | *R. japonica* |  |  |
| 5 |  | *R. japonica* |  |  |
| **1** | VT | ***R. japonica*** | 42.91584 | -72.76998 |
| 2 |  | *R. japonica* |  |  |
| 3 |  | *R. japonica* |  |  |
| 5 |  | *R. japonica* |  |  |
| 1 | VT | *R. japonica* | 43.20189 | -72.45084 |
| 2 |  | *R. japonica* |  |  |
| **3** |  | ***R. japonica*** |  |  |
| 4 |  | *R. japonica* |  |  |
| 5 |  | *R. japonica* |  |  |
| 1 | VT | *R.* × *bohemica* | 43.63969 | -72.32335 |
| 2 |  | *R.* × *bohemica* |  |  |
| **3** |  | ***R.*** × ***bohemica*** |  |  |
| 5 |  | *R. japonica* |  |  |
| 1 | VT | *R. japonica* | 43.84104 | -72.18901 |
| 2 |  | *R. japonica* |  |  |
| **3** |  | ***R. japonica*** |  |  |
| 4 |  | *R. japonica* |  |  |
| 5 |  | *R. japonica* |  |  |
| 1 | NH | *R. japonica* | 43.93716 | -71.68359 |
| 2 |  | *R. japonica* |  |  |
| 3 |  | *R. japonica* |  |  |
| 4 |  | *R. japonica* |  |  |
| **5** |  | ***R. japonica*** |  |  |
| 1 | NH | *R. japonica* | 44.11670 | -71.18184 |
| 2 |  | *R. japonica* |  |  |
| **3** |  | ***R. japonica*** |  |  |
| 4 |  | *R. japonica* |  |  |
| 5 |  | *R. japonica* |  |  |
| 1 | ME | *R. japonica* | 44.11317 | -70.68713 |
| 2 |  | *R. japonica* |  |  |
| 3 |  | *R. japonica* |  |  |
| 4 |  | *R. japonica* |  |  |
| **5** |  | ***R. japonica*** |  |  |
| 1 | ME | *R. japonica* | 44.21219 | -70.30963 |
| 2 |  | *R. japonica* |  |  |
| 3 |  | *R. japonica* |  |  |
| **4** |  | ***R. japonica*** |  |  |
| **3** | ME | ***R. japonica*** | 44.52776 | -69.88677 |
| 4 |  | *R. japonica* |  |  |
| 5 |  | *R. japonica* |  |  |
| 1 | ME | *R.* × *bohemica* | 44.76376 | -69.56170 |
| 2 |  | *R.* × *bohemica* |  |  |
| 3 |  | *R.* × *bohemica* |  |  |
| **4** |  | ***R.*** × ***bohemica*** |  |  |
| 1 | ME | *R. japonica* | 44.93602 | -68.99148 |
| **2** |  | ***R. japonica*** |  |  |
| 3 |  | *R. japonica* |  |  |
| 4 |  | *R. japonica* |  |  |
| 1 | ME | *R. japonica* | 44.94603 | -68.64332 |
| **3** |  | ***R. japonica*** |  |  |
| 4 |  | *R. japonica* |  |  |
| 5 |  | *R. japonica* |  |  |

**Table S2.** Summary of the best models (defined as models with the lowest AICc values) that include latitude fit to data of survival of *S. exigua* to the pupal and adult stages, adult weight of *S. exigua*, and leaf toughness (i.e. variables for which the best overall models included climate; see Table 1). Models of knotweed traits included collection location ID, or collection location ID and shelf for models of herbivore performance, as random intercepts. Significant main and interactive effects (*p* < 0.05) in each model are bolded, and their marginal means or trends calculated using *emmeans* and *emtrends* as discussed in the model interpretation column.

| **Response** | **Best model** | ***R*^2^** | **w_i_** | **Model interpretation** | **Tables** |
| --- | --- | --- | --- | --- | --- |
| Survival (pupa) | None of our top models included latitude |  |  |  | Table S3 |
| Survival (adult) | **Species** + latitude + species * latitude | 0.308 | 0.123 | Survival to adult stage was lower for *R. japonica* than for *R.* × *bohemica* (*p* < 0.001) | Table S4 |
| Adult weight | None of our top models included latitude |  |  |  | Table S6 |
| Leaf toughness | None of our top models included latitude |  |  |  | Table S9 |

**Table S3.** We employed the R function *dredge* to select the best of our generalized linear mixed models (i.e. model with the lowest AICc value) for assessing effects of climate (PC1, PC2, PC3), latitude, and interspecific differences (species) on survival of *S. exigua* to the pupal stage. We fit all models with random intercepts of collection location ID and shelf. *K* refers to the number of estimable parameters in a model. ΔAICc is calculated by subtracting the AICc value of the best model from the AICc of the given model. Akaike weight (w_i_) is the relative likelihood that a given model is the best of a set of models. Only the top models (defined as models with ΔAICc < 2 from the best model; Burnham & Anderson, 2002) are presented (see code for full model set).

| **Model** | ***K*** | **log-lik** | **AICc** | **ΔAICc** | **w_i_** |
| --- | --- | --- | --- | --- | --- |
| Species + PC3 | 5 | -109.723 | 229.7 | 0.00 | 0.438 |
| Species | 4 | -110.875 | 229.9 | 0.21 | 0.395 |
| Species + PC3 + species * PC3 | 6 | -109.632 | 231.7 | 1.94 | 0.166 |

**Table S4.** We employed the R function *dredge* to select the best of our generalized linear mixed models (i.e. model with the lowest AICc value) for assessing effects of climate (PC1, PC2, PC3), latitude, and interspecific differences (species) on survival of *S. exigua* to the adult stage. We fit all models with random intercepts of collection location ID and shelf. *K* refers to the number of estimable parameters in a model. ΔAICc is calculated by subtracting the AICc value of the best model from the AICc of the given model. Akaike weight (w_i_) is the relative likelihood that a given model is the best of a set of models. Only the top models (defined as models with ΔAICc < 2 from the best model; Burnham & Anderson, 2002) are presented (see code for full model set).

| **Model** | ***K*** | **log-lik** | **AICc** | **ΔAICc** | **w_i_** |
| --- | --- | --- | --- | --- | --- |
| Species + PC1 + species * PC1 + PC3 | 7 | -73.574 | 161.7 | 0.00 | 0.159 |
| Species + latitude + species * latitude | 6 | -74.904 | 162.2 | 0.52 | 0.123 |
| Species + PC1 + species * PC1 | 6 | -74.974 | 162.4 | 0.66 | 0.114 |
| Species + PC3 | 5 | -76.117 | 162.5 | 0.83 | 0.105 |
| Species + PC1 + species * PC1 + PC2 | 7 | -74.099 | 162.7 | 1.05 | 0.094 |
| Species + PC1 + species * PC1 + PC2 + species * PC2 | 8 | -73.022 | 162.7 | 1.06 | 0.094 |
| Species + PC1 + species * PC1 + PC2 + PC3 | 8 | -73.104 | 162.9 | 1.22 | 0.087 |
| Species + PC3 + species * PC3 | 6 | -75.346 | 163.1 | 1.40 | 0.079 |
| Species + PC1 + species * PC1 + PC3 + species * PC3 | 8 | -73.199 | 163.1 | 1.41 | 0.079 |
| Species | 4 | -77.632 | 163.5 | 1.76 | 0.066 |

**Table S5.** We employed the R function *dredge* to select the best of our linear mixed models (i.e. model with the lowest AICc value) for assessing effects of climate (PC1, PC2, PC3), latitude, and interspecific differences (species) on pupal weight of *S. exigua*. We fit all models with random intercepts of collection location ID and shelf. *K* refers to the number of estimable parameters in a model. ΔAICc is calculated by subtracting the AICc value of the best model from the AICc of the given model. Akaike weight (w_i_) is the relative likelihood that a given model is the best of a set of models. Only the top models (defined as models with ΔAICc < 2 from the best model; Burnham & Anderson, 2002) are presented (see code for full model set).

| **Model** | ***K*** | **log-lik** | **AICc** | **ΔAICc** | **w_i_** |
| --- | --- | --- | --- | --- | --- |
| Intercept | 4 | 126.082 | -253.0 | 0.00 | 0.728 |
| Species | 5 | 122.014 | -251.0 | 1.97 | 0.272 |

**Table S6.** We employed the R function *dredge* to select the best of our linear mixed models (i.e. model with the lowest AICc value) for assessing effects of climate (PC1, PC2, PC3), latitude, and interspecific differences (species) on adult weight of *S. exigua*. We fit all models with random intercepts of collection location ID and shelf. *K* refers to the number of estimable parameters in a model. ΔAICc is calculated by subtracting the AICc value of the best model from the AICc of the given model. Akaike weight (w_i_) is the relative likelihood that a given model is the best of a set of models. Only the top models (defined as models with ΔAICc < 2 from the best model; Burnham & Anderson, 2002) are presented (see code for full model set).

| **Model** | ***K*** | **log-lik** | **AICc** | **ΔAICc** | **w_i_** |
| --- | --- | --- | --- | --- | --- |
| PC3 | 5 | 74.808 | -157.6 | 0.00 | 0.498 |
| Intercept | 4 | 78.135 | -156.6 | 0.99 | 0.303 |
| PC1 + PC3 | 6 | 70.209 | -155.8 | 1.83 | 0.199 |

**Table S7.** We employed the R function *dredge* to select the best of our linear mixed models (i.e. model with the lowest AICc value) for assessing effects of climate (PC1, PC2, PC3), latitude, and interspecific differences (species) on development time of *S. exigua* to the pupal stage. We fit all models with random intercepts of collection location ID and shelf. *K* refers to the number of estimable parameters in a model. ΔAICc is calculated by subtracting the AICc value of the best model from the AICc of the given model. Akaike weight (w_i_) is the relative likelihood that a given model is the best of a set of models. Only the top models (defined as models with ΔAICc < 2 from the best model; Burnham & Anderson, 2002) are presented (see code for full model set).

| **Model** | ***K*** | **log-lik** | **AICc** | **ΔAICc** | **w_i_** |
| --- | --- | --- | --- | --- | --- |
| Species | 5 | -140.358 | 294.2 | 0.00 | 0.372 |
| Species + latitude | 6 | -136.753 | 295.2 | 1.00 | 0.226 |
| Species + PC1 | 6 | -139.887 | 295.3 | 1.14 | 0.211 |
| Species + PC3 | 6 | -139.563 | 295.5 | 1.32 | 0.192 |

**Table S8.** We employed the R function *dredge* to select the best of our linear mixed models (i.e. model with the lowest AICc value) for assessing effects of climate (PC1, PC2, PC3), latitude, and interspecific differences (species) on development time of *S. exigua* to the adult stage. We fit all models with random intercepts of collection location ID and shelf. *K* refers to the number of estimable parameters in a model. ΔAICc is calculated by subtracting the AICc value of the best model from the AICc of the given model. Akaike weight (w_i_) is the relative likelihood that a given model is the best of a set of models. Only the top models (defined as models with ΔAICc < 2 from the best model; Burnham & Anderson, 2002) are presented (see code for full model set).

| **Model** | ***K*** | **log-lik** | **AICc** | **ΔAICc** | **w_i_** |
| --- | --- | --- | --- | --- | --- |
| Species | 5 | -81.126 | 179.2 | 0.00 | 0.627 |
| Species + PC3 | 6 | -79.184 | 180.3 | 1.04 | 0.373 |

**Table S9.** We employed the R function *dredge* to select the best of our linear mixed models (i.e. model with the lowest AICc value) for assessing effects of climate (PC1, PC2, PC3), latitude, and interspecific differences (species) on leaf toughness. We fit all models with a random intercept of collection location ID. *K* refers to the number of estimable parameters in a model. ΔAICc is calculated by subtracting the AICc value of the best model from the AICc of the given model. Akaike weight (w_i_) is the relative likelihood that a given model is the best of a set of models. Only the top models (defined as models with ΔAICc < 2 from the best model; Burnham & Anderson, 2002) are presented (see code for full model set).

| **Model** | ***K*** | **log-lik** | **AICc** | **ΔAICc** | **w_i_** |
| --- | --- | --- | --- | --- | --- |
| Species + PC1 + PC2 + PC3 + species * PC3 | 8 | -264.108 | 525.2 | 0.00 | 0.458 |
| Species + PC1 + PC2 + species * PC2 + PC3 + species * PC3 | 9 | -264.946 | 527.0 | 1.80 | 0.186 |
| Species + PC1 + PC2 + species * PC1 + PC3 + species * PC3 | 9 | -264.887 | 527.1 | 1.89 | 0.178 |
| Species + PC1 + PC2 + PC3 | 7 | -265.096 | 527.1 | 1.89 | 0.178 |

**Table S10.** We employed the R function *dredge* to select the best of our linear mixed models (i.e. model with the lowest AICc value) for assessing effects of climate (PC1, PC2, PC3), latitude, and interspecific differences (species) on SLA. We fit all models with a random intercept of collection location ID. *K* refers to the number of estimable parameters in a model. ΔAICc is calculated by subtracting the AICc value of the best model from the AICc of the given model. Akaike weight (w_i_) is the relative likelihood that a given model is the best of a set of models. Only the top models (defined as models with ΔAICc < 2 from the best model; Burnham & Anderson, 2002) are presented (see code for full model set).

| **Model** | ***K*** | **log-lik** | **AICc** | **ΔAICc** | **w_i_** |
| --- | --- | --- | --- | --- | --- |
| Species | 4 | 35.716 | -74.1 | 0.00 | 0.219 |
| Species + PC3 | 5 | 33.587 | -74.0 | 0.09 | 0.210 |
| Species + latitude | 5 | 32.249 | -73.4 | 0.68 | 0.156 |
| Species + PC1 + PC3 | 6 | 30.689 | -73.0 | 1.08 | 0.128 |
| Species + PC1 | 5 | 32.663 | -72.8 | 1.32 | 0.113 |
| Species + PC2 + PC3 | 6 | 30.469 | -72.3 | 1.78 | 0.090 |
| Species + PC2 | 5 | 32.487 | -72.2 | 1.91 | 0.084 |

**Table S11.** Descriptive statistics (mean, standard deviation, minimum, maximum) for leaf toughness (tough) and SLA among plants within collection locations. Location coordinates (latitude, longitude) and number of plants sampled per location (N) are referenced in Table S1.

| **Location** | **N** | **Latitude** | **Longitude** | **Mean** | | **SD** | | **Minimum** | | **Maximum** | |
| --- | --- | --- | --- | --- | --- | --- | --- | --- | --- | --- | --- |
|  |  |  |  | **Tough** | **SLA** | **Tough** | **SLA** | **Tough** | **SLA** | **Tough** | **SLA** |
| 1 | 5 | 34.24369 | -83.45888 | 6.258 | 13.235 | 0.705 | 4.208 | 5.237 | 9.964 | 7.201 | 20.103 |
| 2 | 5 | 35.10431 | -83.09636 | 6.990 | 10.835 | 0.980 | 0.684 | 5.707 | 10.001 | 8.144 | 11.389 |
| 3 | 5 | 35.46315 | -83.00964 | 7.040 | 11.877 | 0.606 | 1.825 | 6.085 | 10.055 | 7.667 | 14.025 |
| 4 | 5 | 35.73982 | -82.67967 | 6.937 | 12.028 | 0.803 | 1.493 | 6.159 | 10.565 | 7.930 | 14.470 |
| 5 | 5 | 36.07876 | -82.34724 | 6.054 | 13.376 | 1.947 | 2.083 | 4.290 | 10.186 | 9.024 | 15.641 |
| 6 | 5 | 36.21330 | -81.77665 | 6.944 | 11.470 | 0.876 | 1.230 | 6.111 | 9.910 | 8.301 | 12.709 |
| 7 | 4 | 36.37824 | -81.37687 | 6.423 | 11.862 | 0.726 | 1.359 | 5.470 | 9.838 | 7.216 | 12.761 |
| 8 | 5 | 36.64657 | -80.91819 | 6.410 | 10.821 | 0.752 | 0.667 | 5.124 | 9.812 | 7.110 | 11.439 |
| 9 | 5 | 37.13998 | -80.54223 | 5.722 | 12.039 | 0.644 | 1.804 | 5.028 | 10.011 | 6.621 | 14.940 |
| 10 | 6 | 37.27712 | -80.05605 | 5.860 | 12.431 | 0.603 | 1.413 | 5.091 | 10.495 | 6.837 | 14.299 |
| 11 | 2 | 37.36660 | -79.40806 | 5.833 | 15.234 | 0.224 | 0.284 | 5.675 | 15.033 | 5.992 | 15.435 |
| 12 | 5 | 37.42545 | -79.15961 | 5.533 | 16.493 | 0.320 | 2.566 | 5.144 | 13.325 | 5.904 | 19.545 |
| 13 | 5 | 38.06942 | -78.88527 | 5.716 | 13.188 | 0.421 | 1.732 | 5.081 | 11.642 | 6.180 | 15.662 |
| 14 | 4 | 38.45577 | -78.59494 | 6.299 | 10.970 | 0.574 | 1.067 | 5.513 | 10.008 | 6.858 | 12.497 |
| 15 | 5 | 38.64652 | -78.53521 | 6.748 | 10.625 | 0.964 | 0.580 | 5.922 | 9.689 | 8.411 | 11.278 |
| 16 | 5 | 39.08063 | -78.08574 | 6.298 | 12.126 | 0.853 | 3.293 | 5.091 | 9.576 | 7.216 | 17.576 |
| 17 | 4 | 39.27235 | -77.54727 | 5.390 | 14.801 | 0.203 | 1.849 | 5.108 | 12.710 | 5.578 | 17.193 |
| 18 | 5 | 39.61071 | -76.68270 | 6.182 | 11.737 | 0.376 | 2.175 | 5.783 | 9.576 | 6.605 | 15.319 |
| 19 | 3 | 39.79606 | -76.73519 | 5.914 | 13.038 | 0.201 | 2.252 | 5.743 | 11.560 | 6.136 | 15.631 |
| 20 | 5 | 40.09314 | -76.67698 | 5.136 | 16.722 | 0.901 | 3.580 | 4.321 | 11.543 | 6.541 | 21.475 |
| 21 | 5 | 40.16614 | -76.29577 | 4.911 | 17.939 | 0.751 | 4.749 | 3.612 | 12.342 | 5.390 | 23.352 |
| 22 | 5 | 40.28092 | -75.94827 | 4.500 | 17.689 | 0.576 | 3.923 | 3.870 | 12.625 | 5.126 | 23.529 |
| 23 | 5 | 40.34566 | -75.48387 | 5.528 | 18.463 | 0.801 | 4.858 | 4.748 | 14.688 | 6.669 | 26.931 |
| 24 | 5 | 40.63584 | -75.39360 | 6.252 | 13.483 | 0.644 | 2.028 | 5.502 | 11.225 | 6.974 | 16.177 |
| 25 | 5 | 40.73400 | -75.08229 | 5.158 | 19.255 | 1.617 | 11.142 | 3.401 | 10.880 | 6.652 | 38.578 |
| 26 | 5 | 40.80224 | -74.69688 | 6.527 | 11.619 | 0.280 | 1.166 | 6.183 | 10.593 | 6.861 | 12.899 |
| 27 | 5 | 41.01127 | -74.35119 | 5.169 | 14.661 | 0.553 | 2.944 | 4.336 | 11.419 | 5.822 | 18.004 |
| 28 | 5 | 41.20022 | -74.19431 | 5.674 | 15.010 | 0.751 | 1.788 | 4.598 | 13.476 | 6.453 | 17.870 |
| 29 | 5 | 41.41457 | -73.95776 | 5.828 | 13.000 | 0.475 | 3.242 | 5.134 | 9.828 | 6.428 | 18.387 |
| 30 | 5 | 41.76539 | -73.86471 | 6.599 | 12.500 | 0.963 | 1.645 | 5.537 | 10.531 | 7.496 | 14.014 |
| 31 | 4 | 41.95216 | -73.51009 | 4.615 | 22.250 | 0.401 | 7.808 | 4.086 | 15.067 | 5.052 | 33.348 |
| 32 | 4 | 42.28011 | -73.29808 | 5.327 | 21.020 | 0.734 | 7.132 | 4.423 | 15.066 | 5.941 | 31.273 |
| 33 | 5 | 42.38676 | -72.53005 | 5.970 | 13.271 | 0.568 | 1.523 | 5.248 | 11.490 | 6.595 | 14.983 |
| 34 | 5 | 42.63896 | -72.91260 | 5.862 | 11.857 | 0.549 | 1.131 | 5.057 | 10.651 | 6.481 | 13.170 |
| 35 | 4 | 42.91584 | -72.76998 | 5.702 | 12.334 | 0.151 | 0.483 | 5.480 | 11.756 | 5.815 | 12.938 |
| 36 | 5 | 43.20189 | -72.45084 | 5.815 | 12.608 | 0.391 | 1.521 | 5.217 | 10.476 | 6.276 | 14.405 |
| 37 | 4 | 43.63969 | -72.32335 | 5.459 | 12.661 | 0.521 | 0.617 | 5.026 | 11.979 | 6.096 | 13.373 |
| 38 | 5 | 43.84104 | -72.18901 | 6.051 | 14.047 | 1.341 | 3.985 | 4.784 | 10.222 | 7.793 | 19.118 |
| 39 | 5 | 43.93716 | -71.68359 | 5.897 | 12.134 | 0.670 | 1.288 | 4.858 | 11.300 | 6.729 | 14.387 |
| 40 | 5 | 44.11670 | -71.18184 | 6.385 | 12.207 | 0.708 | 0.817 | 5.415 | 11.078 | 7.216 | 13.050 |
| 41 | 5 | 44.11317 | -70.68713 | 6.020 | 13.008 | 0.822 | 1.932 | 5.263 | 10.729 | 7.272 | 15.453 |
| 42 | 4 | 44.21219 | -70.30963 | 6.632 | 12.761 | 0.762 | 2.214 | 5.910 | 9.506 | 7.705 | 14.466 |
| 43 | 3 | 44.52776 | -69.88677 | 6.047 | 11.270 | 0.244 | 0.870 | 5.804 | 10.272 | 6.291 | 11.866 |
| 44 | 4 | 44.76376 | -69.56170 | 4.876 | 14.892 | 0.775 | 3.244 | 3.728 | 12.243 | 5.395 | 19.371 |
| 45 | 4 | 44.93602 | -68.99148 | 6.275 | 13.590 | 1.351 | 3.457 | 4.773 | 11.140 | 7.906 | 18.608 |
| 46 | 4 | 44.94603 | -68.64332 | 5.627 | 13.084 | 0.562 | 2.002 | 5.031 | 11.351 | 6.175 | 15.513 |

**Table S12.** Bioclimatic variable loadings on PC1, PC2, and PC3 (see Figure S2).

| **PC** | **bio2** | **bio7** | **bio8** | **bio9** | **bio10** | **bio12** | **bio15** |
| --- | --- | --- | --- | --- | --- | --- | --- |
| PC1 | -0.2379419 | 0.5727485 | -0.1108024 | -0.5329579 | -0.4331006 | -0.3459587 | -0.1084341 |
| PC2 | -0.05814795 | 0.02126383 | -0.56858668 | 0.20928917 | -0.37336337 | 0.54129416 | -0.44347978 |
| PC3 | -0.69572624 | -0.19949604 | 0.33496339 | -0.10578793 | 0.24969005 | 0.05922408 | -0.53565115 |

**Table S13.** We employed the R function *dredge* to select the best of our generalized linear mixed models (i.e. model with the lowest AICc value) for assessing effects of climate (PC2 and PC3), latitude (instead of climate PC1), and interspecific differences (species) on survival of *S. exigua* to the pupal stage. We fit all models with random intercepts of collection location ID and shelf. *K* refers to the number of estimable parameters in a model. ΔAICc is calculated by subtracting the AICc value of the best model from the AICc of the given model. Akaike weight (w_i_) is the relative likelihood that a given model is the best of a set of models. Only the top models (defined as models with ΔAICc < 2 from the best model; Burnham & Anderson, 2002) are presented (see code for full model set). Significant main and interactive effects (*p* < 0.05) in the best model are bolded.

| **Model** | ***K*** | **log-lik** | **AICc** | **ΔAICc** | **w_i_** |
| --- | --- | --- | --- | --- | --- |
| **Species** + PC3 | 5 | -109.723 | 229.7 | 0.00 | 0.438 |
| Species | 4 | -110.875 | 229.9 | 0.21 | 0.395 |
| Species + PC3 + species * PC3 | 6 | -109.632 | 231.7 | 1.94 | 0.166 |

**Table S14.** We employed the R function *dredge* to select the best of our generalized linear mixed models (i.e. model with the lowest AICc value) for assessing effects of climate (PC2 and PC3), latitude (instead of climate PC1), and interspecific differences (species) on survival of *S. exigua* to the adult stage. We fit all models with random intercepts of collection location ID and shelf. *K* refers to the number of estimable parameters in a model. ΔAICc is calculated by subtracting the AICc value of the best model from the AICc of the given model. Akaike weight (w_i_) is the relative likelihood that a given model is the best of a set of models. Only the top models (defined as models with ΔAICc < 2 from the best model; Burnham & Anderson, 2002) are presented (see code for full model set). Significant main and interactive effects (*p* < 0.05) in the best model are bolded.

| **Model** | ***K*** | **log-lik** | **AICc** | **ΔAICc** | **w_i_** |
| --- | --- | --- | --- | --- | --- |
| **Species** + latitude + **species** * **latitude** + PC3 | 7 | -73.603 | 161.8 | 0.00 | 0.153 |
| Species + latitude + species * latitude + PC2 + species * PC2 | 8 | -72.734 | 162.2 | 0.42 | 0.124 |
| Species + latitude + species * latitude | 6 | -74.904 | 162.2 | 0.46 | 0.121 |
| Species + latitude + species * latitude + PC2 | 7 | -73.897 | 162.3 | 0.59 | 0.114 |
| Species + PC3 | 5 | -76.117 | 162.5 | 0.77 | 0.104 |
| Species + latitude + species * latitude + PC2 + PC3 | 8 | -72.971 | 162.6 | 0.89 | 0.098 |
| Species + latitude + species * latitude + PC3 + species * PC3 | 8 | -73.189 | 163.1 | 1.33 | 0.079 |
| Species + PC3 + species * PC3 | 6 | -75.346 | 163.1 | 1.35 | 0.078 |
| Species + latitude + species * latitude + PC2 + species * PC2 + PC3 | 9 | -72.280 | 163.4 | 1.69 | 0.065 |
| Species | 4 | -77.632 | 163.5 | 1.70 | 0.065 |

**Table S15.** We employed the R function *dredge* to select the best of our linear mixed models (i.e. model with the lowest AICc value) for assessing effects of climate (PC2 and PC3), latitude (instead of climate PC1), and interspecific differences (species) on pupal weight of *S. exigua*. We fit all models with random intercepts of collection location ID and shelf. *K* refers to the number of estimable parameters in a model. ΔAICc is calculated by subtracting the AICc value of the best model from the AICc of the given model. Akaike weight (w_i_) is the relative likelihood that a given model is the best of a set of models. Only the top models (defined as models with ΔAICc < 2 from the best model; Burnham & Anderson, 2002) are presented (see code for full model set). Significant main and interactive effects (*p* < 0.05) in the best model are bolded.

| **Model** | ***K*** | **log-lik** | **AICc** | **ΔAICc** | **w_i_** |
| --- | --- | --- | --- | --- | --- |
| Intercept | 4 | 126.082 | -253.0 | 0.00 | 0.728 |
| Species | 5 | 122.014 | -251.0 | 1.97 | 0.272 |

**Table S16.** We employed the R function *dredge* to select the best of our linear mixed models (i.e. model with the lowest AICc value) for assessing effects of climate (PC2 and PC3), latitude (instead of climate PC1), and interspecific differences (species) on adult weight of *S. exigua*. We fit all models with random intercepts of collection location ID and shelf. *K* refers to the number of estimable parameters in a model. ΔAICc is calculated by subtracting the AICc value of the best model from the AICc of the given model. Akaike weight (w_i_) is the relative likelihood that a given model is the best of a set of models. Only the top models (defined as models with ΔAICc < 2 from the best model; Burnham & Anderson, 2002) are presented (see code for full model set). Significant main and interactive effects (*p* < 0.05) in the best model are bolded.

| **Model** | ***K*** | **log-lik** | **AICc** | **ΔAICc** | **w_i_** |
| --- | --- | --- | --- | --- | --- |
| PC3 | 5 | 74.808 | -157.6 | 0.00 | 0.494 |
| Intercept | 4 | 78.135 | -156.6 | 0.99 | 0.301 |
| Latitude + PC3 | 6 | 73.184 | -155.9 | 1.76 | 0.205 |

**Table S17.** We employed the R function *dredge* to select the best of our linear mixed models (i.e. model with the lowest AICc value) for assessing effects of climate (PC2 and PC3), latitude (instead of climate PC1), and interspecific differences (species) on development time of *S. exigua* to the pupal stage. We fit all models with random intercepts of collection location ID and shelf. *K* refers to the number of estimable parameters in a model. ΔAICc is calculated by subtracting the AICc value of the best model from the AICc of the given model. Akaike weight (w_i_) is the relative likelihood that a given model is the best of a set of models. Only the top models (defined as models with ΔAICc < 2 from the best model; Burnham & Anderson, 2002) are presented (see code for full model set). Significant main and interactive effects (*p* < 0.05) in the best model are bolded.

| **Model** | ***K*** | **log-lik** | **AICc** | **ΔAICc** | **w_i_** |
| --- | --- | --- | --- | --- | --- |
| **Species** | 5 | -140.358 | 294.2 | 0.00 | 0.471 |
| Species + latitude | 6 | -136.753 | 295.2 | 1.00 | 0.286 |
| Species + PC3 | 6 | -139.563 | 295.5 | 1.32 | 0.243 |

**Table S18.** We employed the R function *dredge* to select the best of our linear mixed models (i.e. model with the lowest AICc value) for assessing effects of climate (PC2 and PC3), latitude (instead of climate PC1), and interspecific differences (species) on development time of *S. exigua* to the adult stage. We fit all models with random intercepts of collection location ID and shelf. *K* refers to the number of estimable parameters in a model. ΔAICc is calculated by subtracting the AICc value of the best model from the AICc of the given model. Akaike weight (w_i_) is the relative likelihood that a given model is the best of a set of models. Only the top models (defined as models with ΔAICc < 2 from the best model; Burnham & Anderson, 2002) are presented (see code for full model set). Significant main and interactive effects (*p* < 0.05) in the best model are bolded.

| **Model** | ***K*** | **log-lik** | **AICc** | **ΔAICc** | **w_i_** |
| --- | --- | --- | --- | --- | --- |
| **Species** | 5 | -81.126 | 179.2 | 0.00 | 0.627 |
| Species + PC3 | 6 | -79.184 | 180.3 | 1.04 | 0.373 |

**Table S19.** We employed the R function *dredge* to select the best of our linear mixed models (i.e. model with the lowest AICc value) for assessing effects of climate (PC2 and PC3), latitude (instead of climate PC1), and interspecific differences (species) on leaf toughness. We fit all models with a random intercept of collection location ID. *K* refers to the number of estimable parameters in a model. ΔAICc is calculated by subtracting the AICc value of the best model from the AICc of the given model. Akaike weight (w_i_) is the relative likelihood that a given model is the best of a set of models. Only the top models (defined as models with ΔAICc < 2 from the best model; Burnham & Anderson, 2002) are presented (see code for full model set). Significant main and interactive effects (*p* < 0.05) in the best model are bolded.

| **Model** | ***K*** | **log-lik** | **AICc** | **ΔAICc** | **w_i_** |
| --- | --- | --- | --- | --- | --- |
| **Species** + **latitude** + **PC2** + PC3 + **species** * **PC3** | 8 | -264.480 | 524.4 | 0.00 | 0.555 |
| Species + latitude + PC2 + species * latitude + PC3 + species * PC3 | 9 | -265.900 | 526.1 | 1.70 | 0.238 |
| Species + latitude + PC2 + species * PC2 + PC3 + species * PC3 | 9 | -265.398 | 526.4 | 1.97 | 0.207 |

**Table S20.** We employed the R function *dredge* to select the best of our linear mixed models (i.e. model with the lowest AICc value) for assessing effects of climate (PC2 and PC3), latitude (instead of climate PC1), and interspecific differences (species) on SLA. We fit all models with a random intercept of collection location ID. *K* refers to the number of estimable parameters in a model. ΔAICc is calculated by subtracting the AICc value of the best model from the AICc of the given model. Akaike weight (w_i_) is the relative likelihood that a given model is the best of a set of models. Only the top models (defined as models with ΔAICc < 2 from the best model; Burnham & Anderson, 2002) are presented (see code for full model set). Significant main and interactive effects (*p* < 0.05) in the best model are bolded.

| **Model** | ***K*** | **log-lik** | **AICc** | **ΔAICc** | **w_i_** |
| --- | --- | --- | --- | --- | --- |
| **Species** | 4 | 35.716 | -74.1 | 0.00 | 0.207 |
| Species + PC3 | 5 | 33.587 | -74.0 | 0.09 | 0.198 |
| Species + latitude + PC3 | 6 | 30.337 | -73.8 | 0.29 | 0.179 |
| Species + latitude | 5 | 32.249 | -73.4 | 0.68 | 0.147 |
| Species + latitude + PC2 + PC3 | 7 | 27.529 | -72.7 | 1.36 | 0.105 |
| Species + PC2 + PC3 | 6 | 30.469 | -72.3 | 1.78 | 0.085 |
| Species + PC2 | 5 | 32.487 | -72.2 | 1.91 | 0.080 |

**Table S21.** Pairwise comparisons for significant main and interactive effects in the best models fit to data of herbivore performance and knotweed leaf traits. Significant main and interactive effects (*p* < 0.05) are bolded. An odds ratio greater than one indicates that the first group has a higher likelihood of surviving than the second group. A positive mean difference or ratio (SLA) greater than one indicates that the first group has a higher estimate than the second group. PC1, PC2, and PC3 represent the three linear principal components (PCs) for climate.

| **Response** | **Best model** | **Groups compared** | **Estimate** | **SE** | **df** | ***p*** |
| --- | --- | --- | --- | --- | --- | --- |
| Survival (pupa) | **Species** + PC3 | *R.* × *bohemica* / *R. japonica* | 5.09 | 2.35 | Inf | 0.0004 |
| Survival (adult) | **Species** + PC1 + **species** * **PC1** + PC3 | *R.* × *bohemica*: Low PC1 / High PC1  *R. japonica*: Low PC1 / High PC1 | 3.15  0.0569 | 3.37  0.0941 | Inf  Inf | 0.2825  0.0831 |
| Pupal weight | ~1 (intercept-only model) |  |  |  |  |  |
| Adult weight | PC3 |  |  |  |  |  |
| Development time to pupal stage | **Species** | *R.* × *bohemica* - *R. japonica* | -5.26 | 1.05 | 22.5 | <0.0001 |
| Development time to adult stage | **Species** | *R.* × *bohemica* - *R. japonica* | -5.59 | 1.85 | 16.9 | 0.0078 |
| Leaf toughness | **Species** + **PC1** + **PC2** + PC3 + species * PC3 | *R.* × *bohemica* - *R. japonica*  Low PC1 - High PC1  Low PC2 - High PC2 | -0.731  0.922  -0.775 | 0.157  0.303  0.363 | 63.3  39.1  39 | <0.0001  0.0042  0.0391 |
| SLA | **Species** | *R.* × *bohemica* / *R. japonica* | 1.25 | 0.0471 | 65.9 | <0.0001 |
